# A novel bacterial thiosulfate oxidation pathway provides a new clue about the formation of zero-valent sulfur in deep sea

**DOI:** 10.1038/s41396-020-0684-5

**Published:** 2020-05-26

**Authors:** Jing Zhang, Rui Liu, Shichuan Xi, Ruining Cai, Xin Zhang, Chaomin Sun

**Affiliations:** 1 CAS Key Laboratory of Experimental Marine Biology & Center of Deep Sea Research, Institute of Oceanology, Chinese Academy of Sciences, Qingdao, China; 2 Laboratory for Marine Biology and Biotechnology, Qingdao National Laboratory for Marine Science and Technology, Qingdao, China; 3 College of Earth Science, University of Chinese Academy of Sciences, Beijing, China; 4 Center of Ocean Mega-Science, Chinese Academy of Sciences, Qingdao, China; 5 CAS Key Laboratory of Marine Geology and Environment & Center of Deep Sea Research, Institute of Oceanology, Chinese Academy of Sciences, Qingdao, China

**Keywords:** Water microbiology, Biogeochemistry

## Abstract

Zero-valent sulfur (ZVS) has been shown to be a major sulfur intermediate in the deep-sea cold seep of the South China Sea based on our previous work, however, the microbial contribution to the formation of ZVS in cold seep has remained unclear. Here, we describe a novel thiosulfate oxidation pathway discovered in the deep-sea cold seep bacterium *Erythrobacter flavus* 21–3, which provides a new clue about the formation of ZVS. Electronic microscopy, energy-dispersive, and Raman spectra were used to confirm that *E. flavus* 21–3 effectively converts thiosulfate to ZVS. We next used a combined proteomic and genetic method to identify thiosulfate dehydrogenase (TsdA) and thiosulfohydrolase (SoxB) playing key roles in the conversion of thiosulfate to ZVS. Stoichiometric results of different sulfur intermediates further clarify the function of TsdA in converting thiosulfate to tetrathionate (^−^O_3_S–S–S–SO_3_^−^), SoxB in liberating sulfone from tetrathionate to form ZVS and sulfur dioxygenases (SdoA/SdoB) in oxidizing ZVS to sulfite under some conditions. Notably, homologs of TsdA, SoxB, and SdoA/SdoB widely exist across the bacteria including in *Erythrobacter* species derived from different environments. This strongly indicates that this novel thiosulfate oxidation pathway might be frequently used by microbes and plays an important role in the biogeochemical sulfur cycle in nature.

## Introduction

Zero-valent sulfur (ZVS) is widespread under the sea floor [1], particularly in the cold seep and the hydrothermal system [2–4]. The production of ZVS is an important energy-conserving strategy for prokaryotes inhabiting these areas. Intimately associated with microbial cells, ZVS is regarded as a bio-signature of the activity of sulfur-oxidizing microorganisms [5–7]. The process of ZVS production begins with the formation of polysulfide, which has been implicated in a number of important geochemical reactions, such as pyritization [8]. Cyclooctasulfur S_8_ and inorganic polysulfide (S_n_^2−^) are important forms of ZVS, which is a critical intermediate in the biogeochemical sulfur cycle [9, 10].

As a common substrate oxidized by almost all sulfur lithotrophs, thiosulfate has been suggested to fulfill a key role in the sulfur cycle [11]. At least three thiosulfate oxidation pathways are postulated to exist in sulfur oxidizing bacteria [12, 13], and each has the possibility to form ZVS either extra- or intracellularly. The first is the Sox pathway, which is governed by the conserved operon *SoxXYZABCD* and operates in photo- and chemo-lithotrophic alphaproteobacteria [14, 15]. The oxidation of thiosulfate is performed via a multienzyme complex consisting of the thiosulfate-induced periplasmic proteins SoxAX, SoxYZ, SoxB, and SoxCD. The complete Sox pathway results in sulfate as the sole product, whereas the incomplete pathway (with SoxCD absent) results in either intra- [16–19] or extracellular [20] accumulation of ZVS. However, the colorless sulfur bacteria *Thiomicrospira thermophile*, which contains a complete Sox pathway, exhibits pH-dependent thiosulfate oxidation to either extracellular ZVS and sulfate at low pH (incomplete) or sulfate only at alkaline pH (complete) [21].

The second thiosulfate oxidation pathway is the tetrathionate intermediate (S_4_I) pathway, which involves production and consumption of polythionate [22]. S_4_I is widely found in Beta- and Gammaproteobacteria, particularly the obligate chemolithotrophic genera including *Acidithiobacillus*, *Thiobacillus, Halothiobacillus*, *Thermithiobacillus*, and *Advenella* [23, 24]. The production of tetrathionate is likely coupled with enzymes that subsequently split tetrathionate to thiosulfate, sulfate, or elemental sulfur [25, 26]. In addition, the Betaproteobacteria *Advenella kashmirensis* either oxidizes tetrathionate to sulfate or uses it as an intermediate during thiosulfate oxidation [27].

The third thiosulfate oxidation pathway is branched thiosulfate oxidation, comprehensively characterized in the purple sulfur bacterium *Allochromatium vinosum*, and this pathway involves the interaction of Sox- and S_4_I-related enzyme systems [16, 28]. Under neutral or slightly acidic growth conditions thiosulfate dehydrogenase (TsdA) catalyzes some of the thiosulfate to tetrathionate, while thiosulfate oxidizes to sulfate with the formation of sulfur globules through the Sox system. In conjunction with the dissimilatory sulfite reductase system, sulfur globules are further oxidized to sulfate [29].

Sulfur oxidizing bacteria are defined as microbes that use reductive sulfur compounds as an energy source for growth. They are highly diverse, and include the green and purple sulfur bacteria, purple non-sulfur bacteria and colorless sulfur bacteria. All green sulfur bacteria belong to the phylum Chlorobi and most oxidize sulfide and ZVS to sulfate [20]. Purple sulfur bacteria include the families *Chromatiaceae* and *Ectothiorhodospiraceae* and produce intra- and extracellular sulfur when grown in the presence of sulfide and thiosulfate [29]. Purple non-sulfur bacteria are a highly diverse and heterogeneous group of bacteria [30] that prefer photoheterotrophic growth under anaerobic conditions, and include many species that can grow photoautotrophically with hydrogen or sulfide as the electron donor. The colorless sulfur bacteria are often identified as chemolithoautotrophic, chemolihoheterotrophic, or chemoorganoheterotrophic [31]. The famous genera in this group include *Thiobacillus*, *Acidithiobacillus*, *Halothiobacillus*, and *Beggiatoa* [10, 32].

The genus *Erythrobacter* belongs to the family *Erythrobacteraceae*, members of which have been isolated from seawater, tidal flats, marine sediment, marine invertebrates, and other ecosystems [33]. Family members of *Erythrobacteraceae* offer a valuable source for further studies focused on aerobic anoxygenic phototrophic metabolism [34, 35], carbon cycling in the ocean [36], and have high potential for applications in biotechnology because they contain important enzymes, such as epoxide hydrolase [37, 38] and manganese oxydase [39]. The genus *Erythrobacter* is abundant in different marine environments [40–42] including the deep-sea cold seep where the relative abundance of *Erythrobacter* could reach 4% of the total bacterial community [43], which strongly suggests that *Erythrobacter* plays an important ecological role in the deep-sea cold seep. However, to the best of our knowledge, the involvement of *Erythrobacter* in the oxidation of thiosulfate and other reductive sulfur compounds has not been elucidated.

In our previous study, a large amount of ZVS was found in the chemosynthetic communities of the Formosa Ridge cold seep in the South China Sea, suggesting that ZVS may be a key intermediate in the sulfur cycle in the deep-sea cold seep [4]. However, the microbial contribution to the formation of ZVS was unknown. Here, we report a novel thiosulfate oxidation pathway in *Erythrobacter* (and other bacteria), that provides a new clue about the formation of ZVS in the deep-sea cold seep. The key genes responsible for thiosulfate oxidation and ZVS production in *E. flavus* 21–3 were determined by a combination of proteomic, genetic, and stoichiometric methods. The broad distribution of these genes was investigated and discussed.

## Materials and methods

### Bacterial strains, plasmids, primers, and growth conditions

*E. flavus* 21–3 was isolated from sediment samples collected by RV *KEXUE* from the cold seep in the South China Sea (119°17′04.956′′ E, 22°06′58.384′′N) at a depth of ~1143 m in September 2017 (Supplementary Table S1). The ~0.5 g sediment sample was added to 100 mL of sulfur producing medium (SPM). The SPM contained: 10 g Na_2_S_2_O·5H_2_O, 0.2 g K_2_HPO_4_, 1 g NaHCO_3_, 0.4 g NH_4_Cl, 0.6 g MgSO_4_, 0.001 g FeCl_3_, and 0.001 g MnCl_2_ in 1 L of sea water, and 15 g/L agar was added to make the corresponding solid medium. Samples were incubated at 28 °C with shaking at a speed of 150 rpm for 2 days. For isolation of single colony, gradient dilutions were made before spread plating. Genomic DNA was extracted (water-boiling) from the isolates, and PCR was performed to amplify the 16S rRNA gene sequence as described previously [44]. To determine the phylogenetic position of *E. flavus* 21–3, the 16S rRNA gene sequence was analyzed by the BLAST programs (https://blast.ncbi.nlm.nih.gov/Blast.cgi), and the phylogenetic tree was reconstructed with MEGA X [45]. Pairwise genome comparison using Average Nucleotide Identity (ANI) based on ANIb was performed with PYANI [46].

*E. flavus* 21–3 wild type and the corresponding mutants were cultured at 28 °C in artificial seawater (ASW) marine broth 2216E (5 g tryptone and 1 g yeast extract in 1 L ASW). 1 M thiosulfate stock was added to the autoclaved medium after filtration through 0.22-μm nuclepore track-etched membranes (Whatman, England). The ASW contained: 24.47 g NaCl, 3.917 g Na_2_SO_4_, 0.664 g KCl, 0.024 g SrCl, 4.981 g MgCl·6H_2_O, 1.102 g CaCl_2_, 0.192 g NaHCO_3_, 0.026 g H_3_BO_4_, and 0.0039 g NaF per 1 L of Milli-Q water. The pH was adjusted to between 7.2 and 7.5 using 1 M NaOH. Diluted (tenfold) 2216E medium with or without 40 mM thiosulfate was used to observe the production of extracellular sulfur globules. *Escherichia coli* was grown and maintained at 37 °C in Luria–Bertani (LB) broth or on agar-plates (supplemented with appropriate antibiotic (s) as required). The bacterial strains, plasmids, and primers used in this study are listed in Supplementary Table S2. Antibiotics were added at the following concentrations when necessary: ampicillin, 100 μg/mL; chloramphenicol, 25 μg/mL, and gentamicin, 25 μg/mL.

### Electron microscopic analysis of sulfur globules

Scanning electron microscope (SEM) (S-3400N; Hitachi, Japan), transmission electron microscope (TEM) (HT7700; Hitachi, Japan) and Raman spectra (WITec alpha300 R system; WITec Company, Germany) were used to identify the components and structure of sulfur globules produced by *E. flavus* 21–3. For SEM analysis, a milky white suspension was collected by centrifugation (5000 rpm, 10 min), lyophilized and observed at 5 kV. Energy-Dispersive Spectrum (EDS) (model 550i, IXRF systems, USA) equipment with SEM and TEM was employed at an accelerating voltage of 5 keV for 30 s. The sulfur globules were observed at 80 kV with TEM after being separated by ultracentrifugation according to the method described in Müller et al. [47] with some modifications. Briefly, 10 mL culture of *E. flavus* 21–3 (cultured with 40 mM thiosulfate) was centrifuged twice at 8000 rpm for 20 min, after which the supernatant was centrifuged at 12,000 rpm for 20 min, to remove debris and cells. The supernatant was collected and centrifuged at 31,000 rpm for 2 h at 8 °C in a Beckman SW 40 Ti rotor (California, USA). The pellets were resuspended in 2 mL buffer (10 mM Tris-HCl, 100 mM NaCl, 5 mM CaCl_2_). TEM for the observation of *E. flavus* 21–3 cultured with 40 mM thiosulfate was conducted at 120 kV.

### Proteome analysis

For proteomic profiling, *E. flavus* 21–3 was grown in 100 mL of 2216E medium with or without 40 mM thiosulfate. Cells were harvested after 18 h or 36 h of incubation, and three biological replicates were completed. Cells were harvested by centrifugation at 12,000 rpm for 10 min at 4 °C, and the pellets were washed with 10 mM phosphate buffer solution (PBS pH 7.4), resuspended in lysis buffer (8 M urea, 1% Protease Inhibitor Cocktail) and disrupted by sonication. The remaining debris was removed by centrifugation at 12,000 rpm at 4 °C for 10 min. Finally, the supernatant was collected and the protein concentration was determined with a BCA kit according to the manufacturer’s instructions. Trypsin digestion, TMT labeling, HPLC fractionation, LC-MS/MS analysis, database search and bioinformatics analysis are described in detail in the supplementary information. Analysis of the differentially expressed proteins was performed using HemI software [48].

### Recombinant DNA techniques

Standard methods for DNA manipulation and cloning were used unless otherwise indicated [49]. KOD One^TM^ PCR Master Mix, Ligation high, ReverTra Ace^®^ qPCR RT Master Mix and Realtime PCR Master Mix were obtained from TOYOBO (Osaka, Japan) and used according to the manufacturer’s instructions. Restriction enzymes (FastDigest) were obtained from Thermo Scientific (Waltham, USA). The genotype of the *E. flavus* 21–3 mutant strains generated in this study was confirmed by PCR using the 2× Rapid Taq Master Mix (Vazyme Biotech Co., Ltd, Nanjing, China). Plasmid DNA from *E. coli* was purified using the Insight-ExBio Plasmid Miniprep kit (Qingdao, China). Purification of PCR product was using TIANgel Purification Kit (Beijing, China).

### Construction of *E*. *flavus* 21–3 mutant strains

Homologous recombination techniques were used to generate knockout mutations in *E. flavus* 21–3 according to the method described previously [50] with some modifications. To mutate *tsdA* (locus tag D0Y83_01395), the upstream and downstream flanking regions of the ORF were amplified from the wild type *E. flavus* 21–3 genome using primers tsdA-up_F/tsdA-up_R and tsdA-down_F/tsdA-down_R (Supplementary Table S2), respectively. The upstream and downstream PCR products were purified, combined, and used as templates for an overlap extension PCR of KO-tsdA using primers tsdA-up_F/tsdA-down_R. The KO-tsdA fragment was purified, digested and inserted into the suicide vector pEX-18Gm via *Eco*RΙ and *Bam*HΙ restriction sites. The resulting plasmid (pEX-18Gm-KO-tsdA) was transformed sequentially into *E. coli* SY327 and *E. coli* S17–1 using the CaCl_2_ method. Mating between *E. flavus* 21–3 and *E. coli* S17–1 containing pEX-18Gm-KO-tsdA was cultured at 28 °C for at least 72 h. Single-event recombinant strains were selected on 2216E agar plate supplemented with ampicillin and gentamicin. Colonies containing pEX-18Gm-KO-tsdA were isolated on 2216E agar plates supplemented with 10% sucrose and ampicillin. Putative mutants were checked by PCR with primers tsdA-up_F/tsdA-down_R. The same methods were used to construct other mutant strains using the corresponding primers listed in Supplementary Table S2.

### Analytical techniques for the determination of different sulfur compounds

For the measurement of thiosulfate, sulfate, ZVS and tetrathionate, wild type *E. flavus* 21–3 and corresponding mutants were grown at 28 °C in ASW 2216E supplemented with 40 mM thiosulfate. Concentrations of thiosulfate and sulfate in the medium were measured by iodometric and barium sulfate turbidimetry respectively [51, 52]. Concentrations of tetrathionate were monitored by HPLC (ThermoFisher U3000, Scientific, USA) fitted with a syncronis C18 column (250 mm × 4.6 mm, 5 μm) (Thermo Scientific, USA). The column was eluted with 50 mM KH_2_PO_4_ (pH adjusted to 2.68 with phosphoric acid) at a flow rate of 1.5 mL/min, and UV detection at 215 nm was used to identify product peaks. ZVS (S_8_) was extracted from cultured medium using dichloromethane according to the method described in Houghton et al. [21]. Briefly, 1 mL sample was extracted three times using a total of 5 mL dichloromethane. The extracted dichloromethane was measured on a UV-Vis spectrometer (Infinite M1000 Pro; Tecan, Männedorf, Switzerland) at 270 nm.

### Identification of *tsdA*, *soxB*, *sdoA*, and *sdoB* homologs in bacterial genomes

*TsdA*, *soxB*, *sdoA*, and *sdoB* homologs were identified in bacterial genomes based on BLASTP searches against assembled genomes including archaea and bacteria at Integrated Microbial Genomes (IMG) [53]. Amino acid sequences of TsdA, SoxB, SdoA, and SdoB from *E. flavus* 21–3, TsdA from *A. vinosum* DSM 180 (ADC61061.1), and SoxB from *Paracoccus denitrificans* (CAA55824.2) were used as queries. All positive hits were manually checked for co-occurrence of the *soxYZ* genes. To find homolog genes of *tsdA*, *soxB*, *sdoA* and *sdoB* in the genomes of other *Erythrobacter* (72 assembled genomes, download at May-2019) obtained from National Center for Biotechnology Information (NCBI), a detailed blast was run in Galaxy [54]. All hits used in further analysis had an identity of 40% or higher.

## Results

### Screening, purification, and identification of deep-sea bacteria possessing potential thiosulfate oxidation capability

**Fig. 1:**
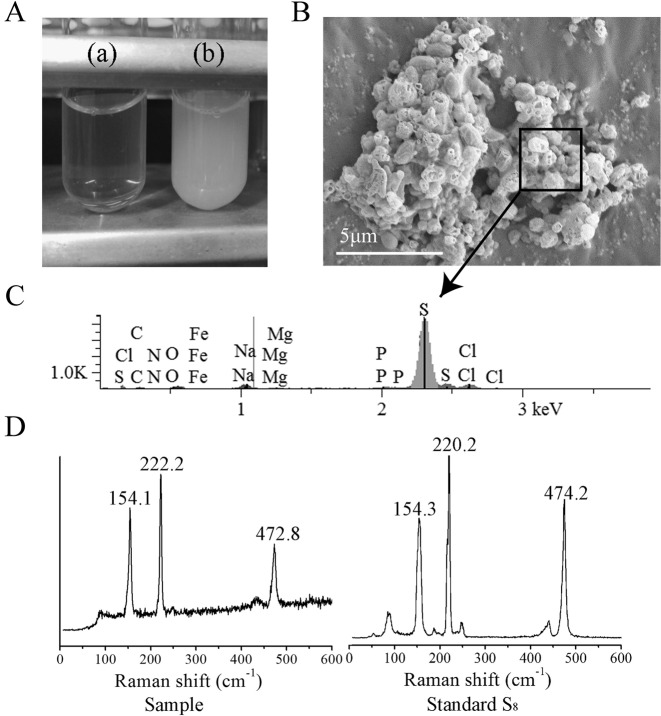
*E. flavus* 21–3 produces ZVS when cultured in medium supplemented with thiosulfate. *E. flavus* 21–3 cultured in diluted 2216E without (**a**) or with 40 mM thiosulfate (**b**). **b** SEM observation of ZVS produced by *E. flavus* 21–3. **c** Energy dispersive spectrum analysis of the selected area in **b**. **d** Raman spectra of the ZVS produced by *E. flavus* 21–3 and standard S_8_.

During the course of screening of deep-sea bacteria possessing potential thiosulfate oxidation capability, strain 21–3 attracted our attention because it produced an obvious milky substance in the culture supplemented with 40 mM thiosulfate after two days of incubation (Fig. 1a), which is a typical indicator of ZVS formation. The cells of strain 21–3 were subsequently purified three times using the dilution-to-extinction technique at 28 °C. The 16S rRNA gene sequence of strain 21–3 (accession no. MN744319) shared a high similarity of 99.93% with *Erythrobacter flavus* SW-46^T^. In addition, strain 21–3 also clustered with *E. flavus* SW-46^T^ according to phylogenetic analysis (Supplementary Fig. S1). The ANI between strain 21–3 and other two strains *E. flavus* VG1 (accession no. ASM223761) and *E. flavus* KJ5 (accession no. ASM429625) was 97.56% and 97.40%, respectively, which were higher than the accepted threshold (ANI value of 94%) for same species [55] Thus, strain 21–3 was identified as a member of *E. flavus* and designated as *E. flavus* 21–3 in this study.

### Identification and characterization of sulfur globules produced by *E. flavus* 21–3 combining with SEM, EDS, Raman spectrum, and TEM

To gain insight into the milky white substance produced by *E. flavus* 21–3 when cultured with thiosulfate, the white substance was subjected to SEM and EDS analyses. SEM results showed that bacterial cells were embedded in globular crystals (Fig. 1b), which is similar to the reported extracellular sulfur globules produced by *Hydrogenovibrio thermophiles*, a chemolithomixotrophic bacterium that uses thiosulfate as an energy source [56]. These crystals were further identified as elemental sulfur by EDS (Fig. 1c). Raman spectrum of the cyclooctasulfur standard was characterized by two strong peaks at 154 and 220 cm^−1^ corresponding to the bending and stretching modes of the eightfold ring and a third peak at 473 cm^−1^ [4, 10]. These typical characteristics of S_8_ were also found in the analysis of the white substance from *E. flavus* 21–3 (Fig. 1d).

**Fig. 2:**
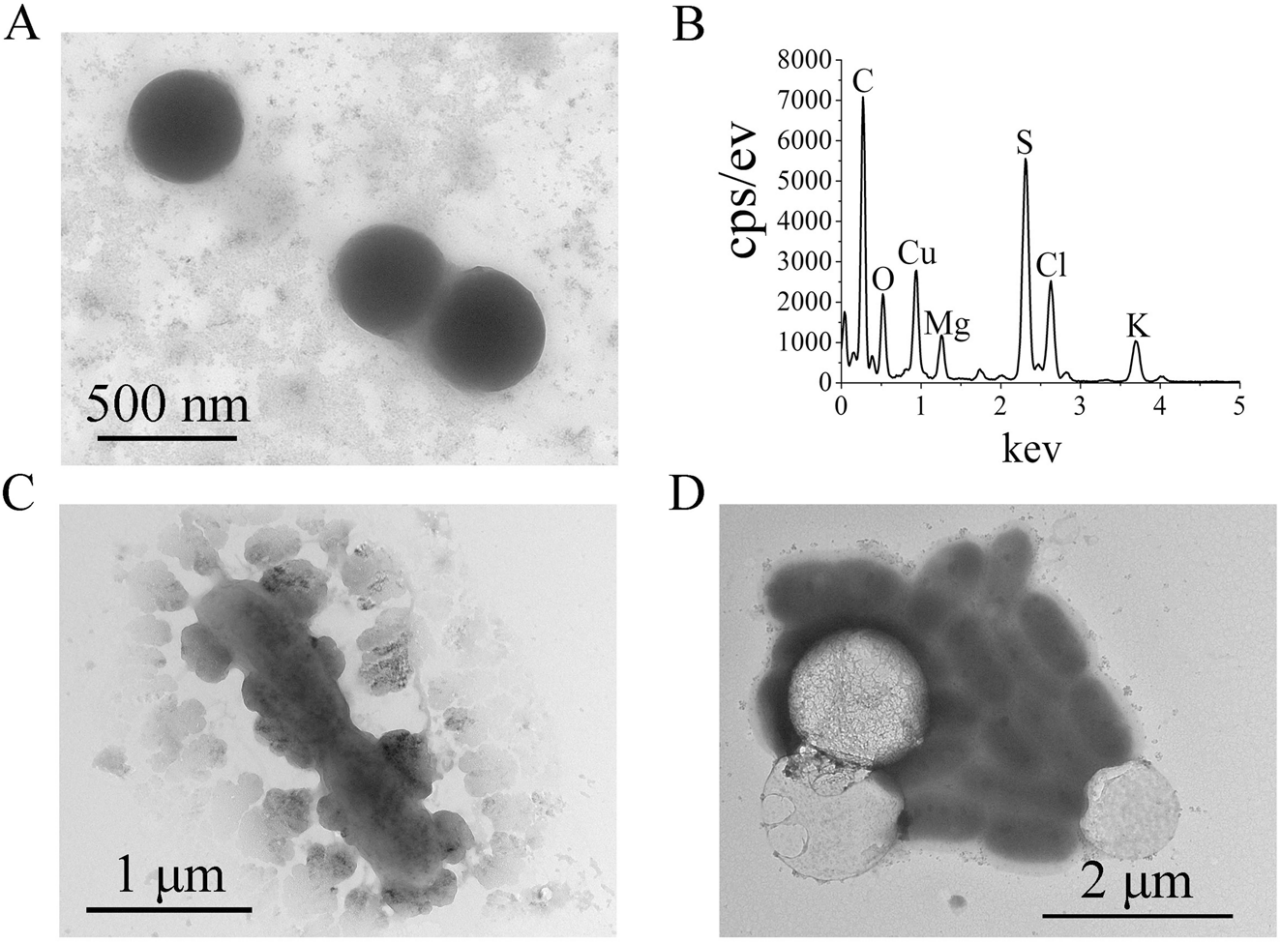
Transmission electron microscopic observation of ZVS produced by *E. flavus* 21–3. **a** TEM image of purified ZVS produced by *E. flavus* 21–3. **b** Energy dispersive spectrum analysis of the separated ZVS shown in panel A. TEM images of *E. flavus* 21–3 cells mixed with ZVS when cultured for 12 h (**c**) or 36 h (**d**) in medium supplemented with 40 mM thiosulfate.

To further characterize the ZVS intermediate formed in *E. flavus* 21–3, sulfur globules produced by the cells were purified and checked with TEM. TEM results showed that the separated sulfur globules (of size 400 nm) were coated with carbon (Fig. 2a) as previously described [57]. TEM-EDS results of the separated sulfur globules (Fig. 2b) were accordant with those of SEM–EDS (Fig. 1c), confirming that *E. flavus* 21–3 is capable to convert thiosulfate to ZVS. TEM was further conducted to observe the production of sulfur globules (S_8_) throughout the growth period of *E. flavus* 21–3 that was cultured with 40 mM thiosulfate. At the start of the early-exponential phase (12 h of incubation) many small solid globules were produced around the cells (Fig. 2c), while at the mid-exponential phase (cultured for 36 h) only a few large, hollow globules were attached to the cells (Fig. 2d). These distinct morphological characteristics of the sulfur globules observed at different growth stages suggest that the sulfur globule may serve as an intermediate during the oxidation of thiosulfate, and indicated that *E. flavus* 21–3 might be a good candidate for the study of microbial thiosulfate oxidation.

### Genomic evidence of thiosulfate oxidation in *E. flavus* 21–3

To obtain a genetic basis of the thiosulfate oxidation in *E. flavus* 21–3, the genome of *E. flavus* 21–3 was completely sequenced using PacBio RSII (Novogene Bioinformatics Technology Co. Ltd, Beijing, China), and the general genomic features are presented in Supplementary Table S3. The complete genome sequence and all genome information of *E. flavus* 21–3 have been deposited to GenBank (accession no. CP032228). When analyzing the *E. flavus* 21–3 genome sequence, we surprised to find only some genes encoding sulfotransferase and thioredoxin-disulfide reductase present, such as disulfide interchange, sulfite exporter, and proteins involved in the metabolism of assimilatory sulfate reduction (Supplementary Table S4). While most typical genes encoding proteins involved in thiosulfate oxidation (the Sox multienzyme complex, flavin cytochrome c-sulfide dehydrogenase, sulfide: quinone oxidoreductase, sulfite: acceptor oxidoreductase, and reverse-acting dissimilatory sulfite reductase) were absent. Given the fact that thiosulfate oxidation occurs in *E. flavus* 21–3, we speculated that a novel pathway may exist in *E. flavus* 21–3.

### Proteomic analyses of thiosulfate oxidation in *E. flavus* 21–3

**Fig. 3:**
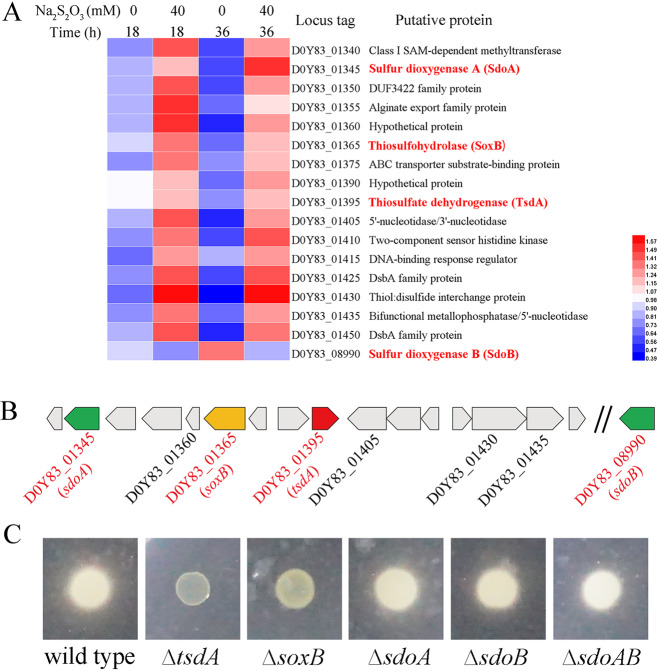
Proteomic and genetic determination of key genes responsible for thiosulfate oxidation in *E. flavus* 21–3. **a** Heatmap analyses of different expressed proteins in *E. flavus* 21–3 when cultured in medium supplemented with 0 or 40 mM thiosulfate after 18 h and 36 h, respectively. Expression of *D0Y83_08990* (*sdoB*) is also shown for its high similarity with *D0Y83_01345* (*sdoA*). **b** Arrangement of the corresponding gene cluster together with *D0Y83_08990* (*sdoB*), possibly involved in thiosulfate oxidation as shown in **a**. **c** Genetic determination of genes involved in thiosulfate oxidation as shown in **a** and **b**. The capacity of *E. flavus* 21–3 wide type and corresponding mutants to produce extracellular sulfur globules was assessed using agar plates supplemented with 40 mM thiosulfate. The formation of a white milky substance around the colonies indicates they are capable of thiosulfate oxidation, while the disappearance of the substance around the colonies indicates this capacity has been lost. The genes responsible for thiosulfate oxidation in *E. flavus* 21–3 (verified by gene disruption) are indicated in red in **a** and **b**.

To better describe the mechanism of thiosulfate oxidation in *E. flavus* 21–3, a proteomic assessment of *E. flavus* 21–3 cultured with or without thiosulfate was performed. The results of this assay showed that 346 of 1068 and 508 of 948 proteins were significantly up-regulated when grown in culture containing thiosulfate at 18 and 36 h, respectively (*P* < 0.05). After carefully analyzing this data, we found that the expression of a gene cluster including genes from *D0Y83_01340* to *D0Y83_01450* (locus tag) was upregulated invariably at both culture time points (Fig. 3a).

Within this gene cluster, the gene *D0Y83_01395* encodes a protein homologous to the c-type cytochrome with C××CH motifs. Since SoxA and TsdA, the typical thiosulfate oxidation proteins, both have the C××CH motif (Supplementary Fig. S2), a phylogenetic analysis was carried out based on the alignment of typical SoxA and TsdA protein sequences. Protein encoded by the gene *D0Y83_01395* clustered with TsdA (Supplementary Fig. S3a), which has been shown to play a key role in the formation of tetrathionate from thiosulfate [58, 59]. Thus, the name *tsdA* was designated to the gene *D0Y83_01395*, with the corresponding protein named TsdA. Given the importance of TsdA in the course of thiosulfate oxidation, we overexpressed and purified the TsdA of *E. flavus* 21–3 in *E. coli*. The apparent molecular mass of purified *E. flavus* 21–3 TsdA is 36 KD (Supplementary Fig. S3b), larger than the homologous protein in *A. vinosum* (27 KD) [60]. The UV–Visible electronic absorption spectrum of the purified recombinant protein has an obvious absorption at 410 nm (Supplementary Fig. S3c), a typical indicator of the existence of cysteine-ligated heme in TsdA [58, 59], which confirms that the *D0Y83_01395*-encoded protein is a homolog of TsdA.

Within the upregulation gene cluster, proteins encoded by genes *D0Y83_01365* and *D0Y83_01435* were both annotated as “bifunctional metallophosphatase/5′-nucleotidases”, and the protein encoded by gene *D0Y83_01405* was annotated as “5′-nucleotidase/3′-nucleotidase”. Further BLASTP results showed that all three proteins possess a conserved domain named “thiosulfohydrolase SoxB”, which is proposed to play a potential role in the cleavage of terminal sulfone groups [61]. The phylogenetic analysis (Supplementary Fig. S4a) and sequence alignment (Supplementary Fig. S4b) of *D0Y83_01365, D0Y83_01405,* and *D0Y83_01435* encoding proteins with other published typical SoxBs further indicate that these three proteins might play SoxB-like function in *E. flavus* 21–3.

The protein encoded by gene *D0Y83_01345* is annotated as “MBL fold metallo-hydrolase” and possesses conserved polysulfide dioxygenase and rhodanase, which are proposed to involve in the oxidation of polysulfide and sulfur [62, 63]. In addition, gene *D0Y83_08990* (beyond this cluster) encodes a protein that is highly similar to the protein encoded by gene *D0Y83_01345* (Supplementary Fig. S5), also suspected to be involved in the oxidation of polysulfide and sulfur. The arrangement of the gene cluster encoding up-regulated proteins, together with gene *D0Y83_08990* is shown in Fig. 3b.

### Genetic determination of key factors responsible for thiosulfate oxidation in *E. flavus* 21–3

Next, we sought to thoroughly investigate the roles of the key genes identified in the proteomic analysis by creating gene knockouts in vivo. After much effort, a genetic operating system was successfully constructed for the first time in the genus *Erythrobacter*. Notably, deletion of *tsdA* (*D0Y83_01395*) and *D0Y83_01365* almost completely disrupted the ability of *E. flavus* 21–3 mutants to form sulfur globules when grown in the presence of thiosulfate (Fig. 3c), which demonstrates that these two genes play key roles in thiosulfate oxidation in *E. flavus* 21–3. Given the close phylogenetic relationship between *D0Y83_01365* encoding protein and other SoxBs (Supplementary Fig. S4a), the gene *D0Y83_01365* was designated as *soxB* with the corresponding protein name SoxB. Though *D0Y83_01405* and *D0Y83_01435* encoding proteins also possess the “thiosulfohydrolase SoxB” domain, deletion of *D0Y83_01405* and *D0Y83_01435* did not affect sulfur globule formation (Supplementary Fig. S6).

Deletion of *D0Y83_01345* and *D0Y83_08990* had no obvious influence on sulfur globule formation (Fig. 3c). However, compared with wild type, the ability of further ZVS oxidation was abolished in the mutant strain *ΔD0Y83_08990*, which suggests a potential function for *D0Y83_08990* encoding protein in ZVS oxidation. Given the high similarity of *D0Y83_01345* and *D0Y83_08990*, we assigned SdoA (sulfur dioxygenase A) and SdoB (sulfur dioxygenase B) to *D0Y83_01345* and *D0Y83_08990* encoding proteins, respectively. Consistently, double mutant strain *ΔsdoAB* showed the same phenotype as that of the mutant strains *ΔsdoA* and *ΔsdoB* (Fig. 3c). The deletion of other genes, including *D0Y83_01360* and *D0Y83_01430*, did not show any effect on thiosulfate oxidation (Supplementary Fig. S6), suggesting they might be not essential for thiosulfate metabolism in *E. flavus* 21–3.

### Thiosulfate oxidation in *E. flavus* 21–3 wild type and mutant strains

**Fig. 4:**
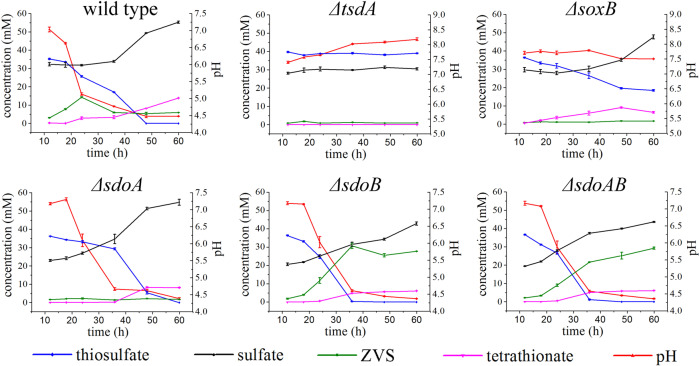
Comparisons of thiosulfate oxidation, generation of sulfate, tetrathionate, and ZVS in *E. flavus* 21–3 wild type and corresponding mutants. The error bars indicate the standard deviation (S.D.) from two different biological replicates.

To reveal the exact function of key genes (*tsdA*, *soxB*, *sdoA*, and *sdoB*) responsible for thiosulfate oxidation determined by proteomic and genetic methods, the stoichiometry of sulfur intermediates in the course of thiosulfate oxidation in *E. flavus* 21–3 wild type and mutants was determined. In wild type *E. flavus* 21–3, the concentration of thiosulfate decreased with the culture time, and reached zero after 48 h of incubation (Fig. 4). Meanwhile, the concentration of sulfate continued to increase throughout the culturing time (~60 h), suggesting that thiosulfate was eventually converted to sulfate. The maximum accumulation of ZVS (S_8_) in the medium appeared at 24 h and then dropped gradually to the detectable amount at the end of culturing time (Fig. 4), while the concentration of the other important intermediate tetrathionate increased slightly throughout the time course of the culture (Fig. 4). The existence of S_8_ and tetrathionate in the medium implies that *E. flavus* 21–3 mediated incomplete oxidation of thiosulfate (Fig. 4). The pH of medium decreased across the culturing time, which strongly indicates the occurrence of sulfone oxidation in the course of thiosulfate oxidation in *E. flavus* 21–3 [61].

Interestingly, the concentration of thiosulfate was constant and tetrathionate was not detected in the *ΔtsdA* mutant culture (Fig. 4). Given that thiosulfate is generally converted to tetrathionate [12], and the fact that tetrathionate was detected in wild type *E. flavus* 21–3 but not in the *ΔtsdA* mutant, it is reasonable to deduce that TsdA mediates the first step of thiosulfate oxidation in this bacterium. It therefore makes sense that neither ZVS nor extra sulfate was detected in the *ΔtsdA* mutant culture medium. In the mutant strain *ΔsoxB*, the concentrations of thiosulfate and tetrathionate gradually decreased and increased respectively, while ZVS was not detected in the medium. Meanwhile, the sulfate concentration was lower in the mutant than that in the wild type, indicating the involvement of SoxB in liberating sulfone from tetrathionate (^−^O_3_S–S–S–SO_3_^−^) during sulfate formation. Taken together, these results suggest that SoxB functions downstream of TsdA but upstream of the key step in ZVS formation.

Mutant strain *ΔsdoA* consumed 40 mM thiosulfate after 60 h of incubation, without obvious accumulation of ZVS. This observation conflicts with the phenotype detected on agar plates (Fig. 3c). However, we also noticed that more ZVS accumulated in the *ΔsdoB* mutant than in the wild type, suggesting that SdoB might act as a key factor in ZVS metabolism. Quantitative real-time PCR analysis showed that expression of *sdoB* was upregulated in the mutant *ΔsdoA* when the bacterium was cultured with 40 mM thiosulfate (Supplementary Fig. S7), which explained the extremely low concentration of ZVS detected in *ΔsdoA*. Consistently, the concentrations of thiosulfate, sulfate, tetrathionate, and ZVS in the double mutant strain *ΔsdoAB* showed similar patterns to those in *ΔsdoB*, confirming the prominent function of SdoB in oxidation of ZVS in *E. flavus* 21–3.

**Fig. 5:**
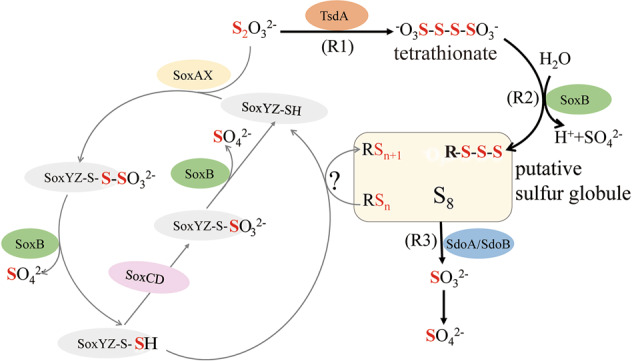
The proposed novel thiosulfate oxidation pathway in *E. flavus* 21–3. Gray lines indicate the classical Sox pathway and black lines represent the novel pathway determined in *E. flavus* 21–3. The classical Sox pathway begins with the combination between thiosulfate and SoxYZ with the help of SoxAX, and results in a thiocysteine-S-sulfate residue (SoxYZ-S-SSO_3_^2−^). Sulfate is liberated by hydrolysis (catalyzed by SoxB) to yield a polysulfide intermediate (SoxYZ-S-S^−^), which is subsequently oxidized by SoxCD to form a cysteine-S sulfate residue (SoxYZ-S-SO_3_^2−^). Hydrolysis by SoxB releases sulfate and regenerates the SoxYZ complex (SoxYZ-SH). In the absence of SoxCD, sulfane splitting from the polysulfide intermediate (SoxYZ-S-S^−^) in an obscure way results in polysulfide and free SoxYZ-SH. The novel pathway found in *E. flavus* 21–3 begins with the formation of tetrathionate from two molecular thiosulfate, catalyzed by TsdA (Reaction 1: R1). SoxB is responsible for liberating the sulfate from tetrathionate (instead of SoxYZ-S-S-SO_3_; Reaction 2: R2). The remaining sulfane is converted to polysulfide, and then relatively stable S_8_. The sulfur globule is finally oxidized by SdoA/SdoB, with sulfite as the intermediate product (Reaction 3: R3). Sulfite is non-enzymatically/enzymatically converted to sulfate. SoxAX sulfur oxidation c-type cytochrome, SoxB thiosulfohydrolase, SoxYZ thiosulfate oxidation carrier protein, SoxCD sulfur oxidation protein. TsdA thiosulfate dehydrogenase, SdoA/SdoB sulfur dioxygenase A/sulfur dioxygenase B.

Based on the combination of proteomic, genetic, and stoichiometric data on thiosulfate oxidation in *E. flavus* 21–3 we propose a novel thiosulfate oxidation pathway (Fig. 5). Briefly, thiosulfate oxidation in *E. flavus* 21–3 is initially mediated by TsdA, which converts thiosulfate to tetrathionate [58, 59]. Tetrathionate is further hydrolyzed by SoxB to form sulfate and release H^+^, which decreases the environment pH. The remaining sulfur atoms may attach to membrane-bound thiol groups or be added to other polysulfide species [10]. However, polysulfide (polythionate) is unstable in acidic conditions and is therefore partly converted to stable cyclooctasulfur S_8_ [8, 64, 65], forming the putative sulfur globules. In the presence of SdoA/SdoB, sulfur globules are further oxidized to sulfite and then non-enzymatically/enzymatically converted to sulfate.

### Wide distribution of the novel thiosulfate oxidation pathway determined in *E. flavus* 21–3

**Fig. 6:**
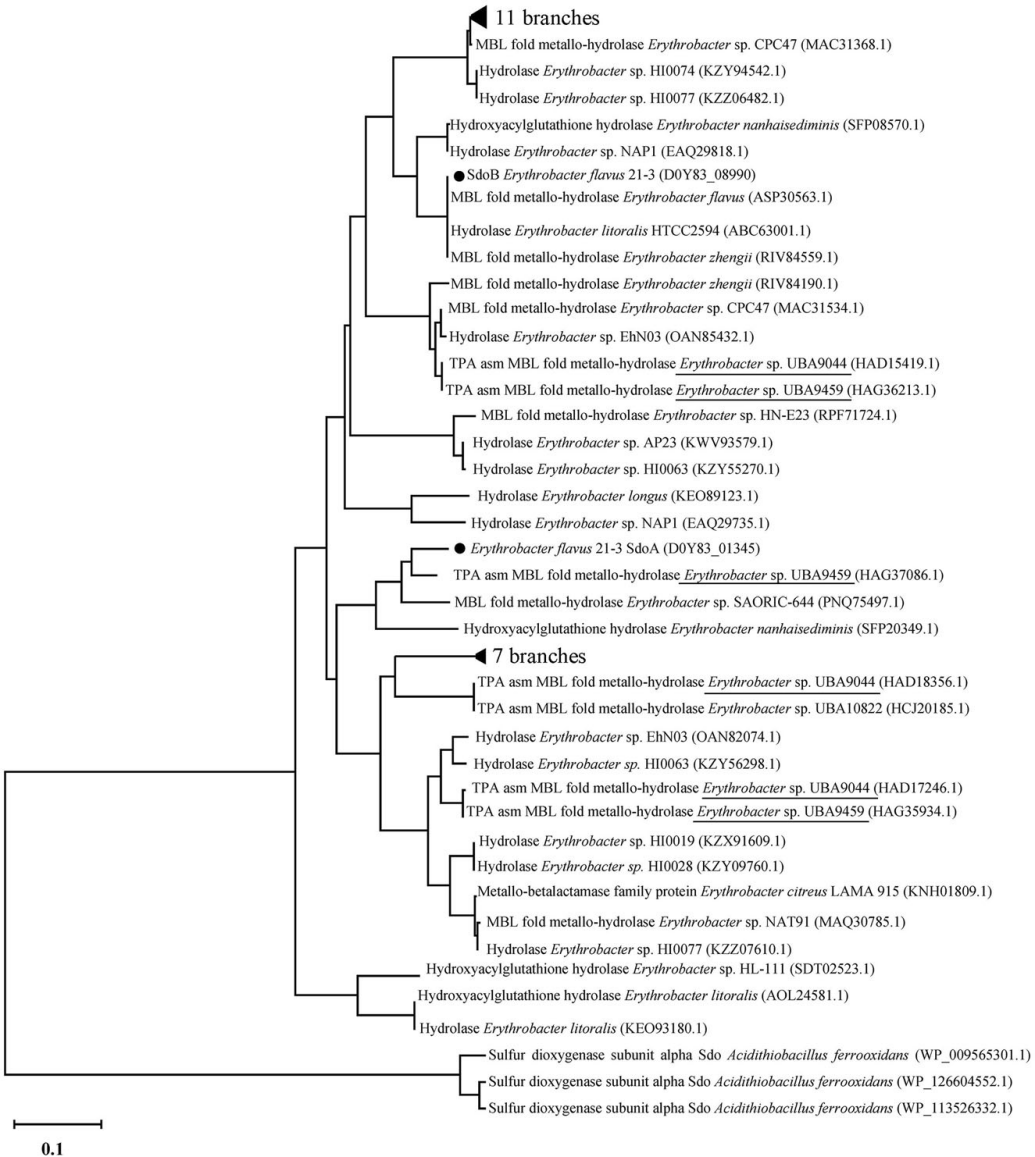
The consensus phylogenetic tree of SdoA and SdoB in *E. flavus* 21–3 with other related SdoA/SdoB obtained from assembled genomes of *Erythrobacter* and *Acidithiobacillus* (accession numbers are indicated after each species name), constructed by the neighbor-joining method. Sequences from *Erythrobacter* sp. UBA9044 and *Erythrobacter* sp. UBA9459 are underlined.

To explore homologs of TsdA, SoxB, and SdoA/SdoB in other *Erythrobacter* species, we searched all assembled genomes of *Erythrobacter* in the NCBI database. Protein sequences PHR02631.1, HAG37069.1, and HAD16915.1 in the *Erythrobacter* genomes of NORP101, UBA9459, and UBA9044, respectively, showed 99.9% identity with TsdA, and C××CH motifs were also identified in these sequences (Supplementary Fig. S8). Protein sequences HAG37075.1 and HAD16921.1 in the *Erythrobacter* genomes of UBA9459 and UBA9044 showed 97% identity with SoxB (Supplementary Fig. S9). SdoA/SdoB homologs were found to exist in half of the assembled genomes of *Erythrobacter*, including in UBA9459 and UBA9044. A phylogenic tree was constructed using SdoA/SdoB sequences from both *Erythrobacter* and *Acidithiobacillus* (the typical bacterium possessing ZVS oxidation) (Fig. 6). The results showed that SdoA and SdoB from *Erythrobacter* were clustered into a separate clade with those from *Acidithiobacillus*. Given the specific function of SdoA/SdoB in ZVS oxidation, the resulting sequences clustered with SdoA/SdoB were expected to have the same function. Overall, our analysis revealed that homologous sequences of TsdA, SoxB, and SdoA/SdoB were found in assembled *Erythrobacter* genomes UBA9459 and UBA9044, indicating that this novel thiosulfate oxidation pathway might exist in other *Erythrobacter* species.

**Fig. 7:**
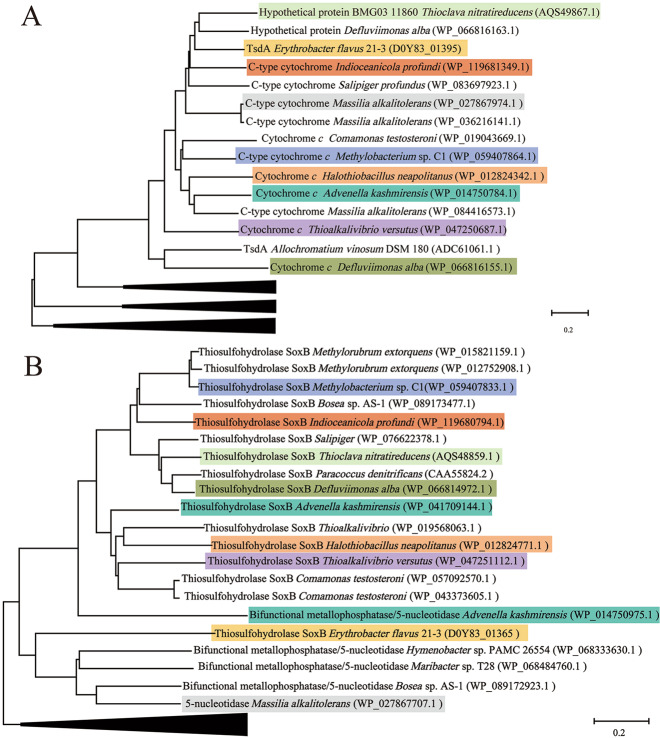
Phylogenetic analysis of TsdA and SoxB homologs. The consensus phylogenetic tree of TsdA (**a**) and SoxB (**b**) in *E. flavus* 21–3 with other homologs obtained from public bacterial genomes (accession numbers are indicated after each species name) constructed by the neighbor-joining method. Branches shown in the tree represent sequences clustered with typical TsdA or SoxB, and each color indicates a specific species in **a** and **b**. Related species containing both TsdA and SoxB and their corresponding GenBank accession numbers are listed as follows: *Advenella kashmirensis*: GCA_000219915.3; *Methylobacterium* sp. C1: GCA_001854385.1; *Indioceanicola profundi*: GCA_003568845.1; *Thioclava nitratireducens*: GCA_000024765.1; *Defluviimonas alba*: GCA_000024765.1; *Halothiobacillus neapolitanus*: GCA_000024765.1; *Thioalkalivibrio versutus*: GCA_001020955.1; *Massilia alkalitolerans*: GCA_000427785.1, and *E. flavus* 21–3: GCA_008932225.1.

Lastly, we checked the bacterial genomes available in IMG database to explore the existence of the novel thiosulfate oxidation pathway in other bacterial species. To ensure the accuracy of this analysis, stringent criteria were applied and only organisms containing both *tsdA* and *soxB* homologous genes were considered. The search results showed that the set of genes were mainly identified in Alphaproteobacteria, Betaproteobacteria, Gammaproteobacteria, and Bacteroidetes (Supplementary Table S5). Moreover, the TsdA and SoxB homologs were also searched in the available metagenomes from IMG. The e-value was set as 1e^−20^ and the sequences with an identity more than 40% were selected. Totally, there were 18 (in total 103) metagenomes containing both TsdA and SoxB homologs (Supplementary Table S6). Notably, the identified bacteria containing both TsdA and SoxB homologs occur naturally in a variety of environments including land soil, acid mine drainage, sea water, and deep-sea sediment. To further clarify the functions of selected TsdA- and SoxB-like proteins, phylogenic trees were constructed using TsdA- and SoxB-like sequences obtained from the identified bacteria (Supplementary Table S5 and Fig. 7). The phylogenetic results showed that these TsdA- or SoxB-like proteins identified in different bacteria clustered with different typical TsdA or SoxB, suggesting that these bacteria might oxidize thiosulfate through the novel pathway. Given the key roles of TsdA and SoxB in this novel pathway and their broad distribution in bacteria, we propose that the novel thiosulfate oxidation pathway identified in *E. flavus* 21–3 exists in many other microorganisms.

## Discussion

Sulfur is a key element whose transformation and status in the environment are critically dependent upon microbial activities. Individual microorganisms have evolved various enzyme systems to catalyze many conversions of sulfur compounds between oxidation states [66]; some sulfur pathways and sulfur-converting enzymes are yet to be discovered and functionally characterized. Our discovery that TsdA and SoxB mediate the conversion of thiosulfate to ZVS in *E. flavus* 21–3 is totally unique compared with the three previously reported thiosulfate oxidation pathways [12], and key proteins associated with this novel pathway are widely distributed in many bacteria. Also, our findings add a new aspect to the current understanding of the source of ZVS in deep-sea cold seep.

Thiosulfate is known to play key roles in the sulfur cycle in marine sediments where it can be oxidized to sulfate by microorganisms in order to harness energy. TsdA and SoxB are two key proteins determined in the novel thiosulfate oxidation pathway we have described here. TsdA is a periplasmic enzyme belonging to the c-type cytochrome family present in a wide range of bacteria [67, 68]. The proposed function of TsdA is to oxidize thiosulfate to tetrathionate; in some case it acts as a bi-functional enzyme to reduce tetrathionate to thiosulfate [68]. In *E. flavus* 21–3, TsdA converts thiosulfate into tetrathionate (Fig. 4), which initiates the subsequent reaction in the novel thiosulfate oxidation pathway. In addition, the gene *D0Y83_01390* located upstream of TsdA encoding gene (*D0Y83_01395*) was proposed to encode a cytochrome *c* family protein TsdB, which might cooperate with TsdA to generate tetrathionate [69]. Tetrathionate is often used by bacteria as an electron acceptor and significantly stimulates the growth of many microbes [68]. Therefore, it is reasonable to propose that *E. flavus* 21–3 might benefit from the oxidation of thiosulfate. Moreover, TsdA is widely distributed among many bacteria (Supplementary Fig. S3), and we predict that this pathway is far more common than currently appreciated.

Tetrathionate is reported to be an intermediate in thiosulfate oxidation in purple sulfur bacteria, and further metabolism of tetrathionate is mediated by tetrathionate hydrolase which converts tetrathionate to thiosulfate, sulfate and sulfur products [25, 26, 70]. Based on the genetic and stoichiometric results from *E. flavus* 21–3 (Figs. 3, 4), we suggest that SoxB hydrolyzes tetrathionate to sulfate and ZVS. This is consistent with a previous study that proposed an essential role for SoxB in tetrathionate oxidation in *A. kashmirensis* [27]. It is worth noting that additional two genes (*D0Y83_01405* and *D0Y83_01435*) in *E. flavus* 21–3 also encode proteins belonging to the 5′-nucleotidase family that show similarity with SoxB (Supplementary Fig. S4a), and these two SoxB-like proteins also contain two kinds of conserved ligands of metal sites as that in typical SoxB (Supplementary Fig. S4B) [61]. We therefore speculate that *D0Y83_01405* and *D0Y83_01435* encoding proteins might hydrolyze tetrathionate and form ZVS and sulfate to some extent in the absent of SoxB. In fact, a small amount of ZVS was observed on agar plates supplemented with thiosulfate in the Δ*soxB* mutant (Fig. 3c), and the concentration of sulfate increased during the 48–60 h along with the decrease of tetrathionate in *ΔsoxB* mutant (Fig. 4). However, the deletion of *D0Y83_01405* and *D0Y83_01435* did not influence the formation of ZVS, which confirms the dominant tetrathionate oxidation function of SoxB in *E. flavus* 21–3.

In *E. flavus* 21–3, ZVS can be further oxidized to sulfite by SdoA/SdoB, in a process strictly limited by the concentration of oxygen [63]. This explains the discrepancy of ZVS formation between agar plates and aerobic liquid medium in *E. flavus* 21–3 wild type and *ΔsoxB* and *ΔsdoA* mutant strains: the concentrations of ZVS decreased after a 24 h incubation of the liquid culture, but remained the same on agar plates throughout the culturing time (Figs. 3c,  4). Sulfite is easily oxidized to sulfate in the presence of oxygen, and this indeed happened in *E. flavus* 21–3 (Figs. 4,  5). There are in fact two steps that would produce sulfate in the course of thiosulfate oxidation in this novel pathway (Fig. 5), and sulfate is no doubt a very important sulfur compound that affects the abundance of sulfate-reducing microorganisms to a great extent [66]. Notably, on a global scale, recent estimates suggest that the remineralisation of up to 29% of the organic matter deposited to the seafloor is facilitated by sulfate-reducing microorganisms [66]. Given the isolation niches of *E. flavus* 21–3 and wide distribution of TsdA/SoxB/SdoA/SdoB in other bacteria, we propose that the contribution of microorganisms possessing this thiosulfate oxidation pathway to the sulfur cycle in the deep-sea cold seep can’t be ignored. Future efforts are required to test the functional role of *E. flavus* 21–3 in thiosulfate oxidation *in situ* and to explore the existence of this novel pathway in other cold-seep bacterial isolates.

### Data deposit

The genome data of *E. flavus* 21–3 have been deposited to the NCBI with the accession number of CP032228. The mass spectrometry proteomics data have been deposited to the Proteome Xchange Consortium via the PRIDE [71] partner repository with the dataset identifier PXD016502.

## Supplementary information

Supplemental data
