# Supplementary material for: A novel bacterial thiosulfate oxidation pathway provides a new clue about the formation of zero-valent sulfur in deep sea
Source: ISME J. 2020 May 26;14(9):2261–74. doi: 10.1038/s41396-020-0684-5 (PMC7608252; doi:10.1038/s41396-020-0684-5)
Supplement: Supplementary file 1 — Supplemental data [file 41396_2020_684_MOESM1_ESM.docx]

**Supplemental data**

**A novel bacterial thiosulfate oxidation pathway provides a new clue about the formation of zero-valent sulfur in deep sea**

Jing Zhang^1,2,3,5^, Rui Liu^1,2,5^, Shichuan Xi^3,4,5^, Ruining Cai^1,2,3,5^, Xin Zhang^4,5^, Chaomin Sun^1,2,5 *^

^1^CAS Key Laboratory of Experimental Marine Biology & Center of Deep Sea Research, Institute of Oceanology, Chinese Academy of Sciences, Qingdao, China

^2^Laboratory for Marine Biology and Biotechnology, Qingdao National Laboratory for Marine Science and Technology, Qingdao, China

^3^College of Earth Science, University of Chinese Academy of Sciences, Beijing, China

^4^CAS Key Laboratory of Marine Geology and Environment & Center of Deep Sea Research, Institute of Oceanology, Chinese Academy of Sciences, Qingdao, China

^5^Center of Ocean Mega-Science, Chinese Academy of Sciences, Qingdao, China

^*^ Corresponding author

Chaomin Sun Tel.: +86 532 82898857; fax: +86 532 82898857.

E-mail address: sunchaomin@qdio.ac.cn

**Supplemental Methods**

**Proteomic profiling: Trypsin digestion, TMT labeling, HPLC fractionation, LC-MS/MS analysis of pepetides.** For digestion, the protein solution was reduced with 5 mM dithiothreitol for 30 min at 56 °C and alkylated with 11 mM iodoacetamide for 15 min at room temperature in darkness. The protein sample was then diluted by adding 100 mM TEAB to urea concentration less than 2M. Finally, trypsin was added at 1:50 trypsin-to-protein mass ratio for the first digestion overnight and 1:100 trypsin-to-protein mass ratio for a second 4 h-digestion. After trypsin digestion, peptide was desalted by Strata X C18 SPE column (Phenomenex) and vacuum-dried. Peptide was reconstituted in 0.5 M TEAB and processed according to the manufacturer’s protocol for TMT kit. The tryptic peptides were fractionated into fractions by high pH reverse-phase HPLC using Agilent 300Extend C18 column (5 μm particles, 4.6 mm ID, 250 mm length). Briefly, peptides were first separated with a gradient of 8% to 32% acetonitrile (pH 9.0) over 60 min into 60 fractions. Then, the peptides were combined into 18 fractions and dried by vacuum centrifuging. The tryptic peptides were dissolved in 0.1% formic acid (solvent A), directly loaded onto a home-made reversed-phase analytical column (15-cm length, 75 μm i.d.). The gradient was comprised of an increase from 6% to 23% solvent B (0.1% formic acid in 98% acetonitrile) over 26 min, 23% to 35% in 8 min and climbing to 80% in 3 min then holding at 80% for the last 3 min, all at a constant flow rate of 400 nL/min on an EASY-nLC 1000 UPLC system. The peptides were subjected to NSI source followed by tandem mass spectrometry (MS/MS) in Q ExactiveTM Plus (Thermo) coupled online to the UPLC. The electrospray voltage applied was 2.0 kV. The m/z scan range was 350 to 1800 for full scan, and intact peptides were detected in the Orbitrap at a resolution of 70,000. Peptides were then selected for MS/MS using NCE setting as 28 and the fragments were detected in the Orbitrap at a resolution of 17,500. A data-dependent procedure that alternated between one MS scan followed by 20 MS/MS scans with 15.0s dynamic exclusion. Automatic gain control (AGC) was set at 5E4. Fixed first mass was set as 100 m/z.

**Database search.** The resulting MS/MS data were processed using Maxquant search engine (v.1.5.2.8). Tandem mass spectra were searched against NCBI database concatenated with reverse decoy database. Trypsin/P was specified as cleavage enzyme allowing up to 2 missing cleavages. The mass tolerance for precursor ions was set as 20 ppm in First search and 5 ppm in Main search, and the mass tolerance for fragment ions was set as 0.02 Da. Carbamidomethyl on Cys was specified as fixed modification and oxidation on Met was specified as variable modifications. FDR was adjusted to < 1% and minimum score for peptides was set > 40.

**Overexpression and** **purification of TsdA.** The ORF encoding *tsdA* gene was amplified from the *E. flavus* 21-3 genome using the primers GTtsdA_F/GTtsdA_R shown in Supplementary Table S1. The PCR product was purified, digested and ligated into the expression vector 2GT [1]. *E. coli* BL21 (DE3) (Novagen) was co-transformed with the resultant construct 2GT-tsdA and a plasmid pEC86, which constitutively expresses the *E. coli* cytochrome C maturation system [2]. Cells were grown aerobically at 37 °C in LB supplemented with ampicillin and chloramphenicol. Protein expression was induced at an OD_600_ around 0.6 with 1 mM isopropyl-1-thio-β-D-galactopyranoside, and the cells were cultured for further 20 h at 16 °C.

Cells were harvested by centrifugation (8,000 rpm, 20 min, 4°C), resuspended in resuspension buffer (20 mM HEPES, pH 7.5, 300 mM KCl, 1 mM DTT, 10 % glycerol) and subjected to sonication. The lysate was centrifuged, filtered and applied to a 5-mL GST column (GE Healthcare). Recombinant TsdA was eluted from the column with elution buffer containing 2.5 mM reduced glutathione. The purity of fractions collected was determined by SDS-PAGE. The GST tag was removed by TEV protease [3] through dialysis to TEV cleavage buffer (50 mM Tris, 150 mM NaCl, 10% glycerol, pH 7.5), protein aliquots were stored in -80 °C till future use.

**Supplementary Figures**

**
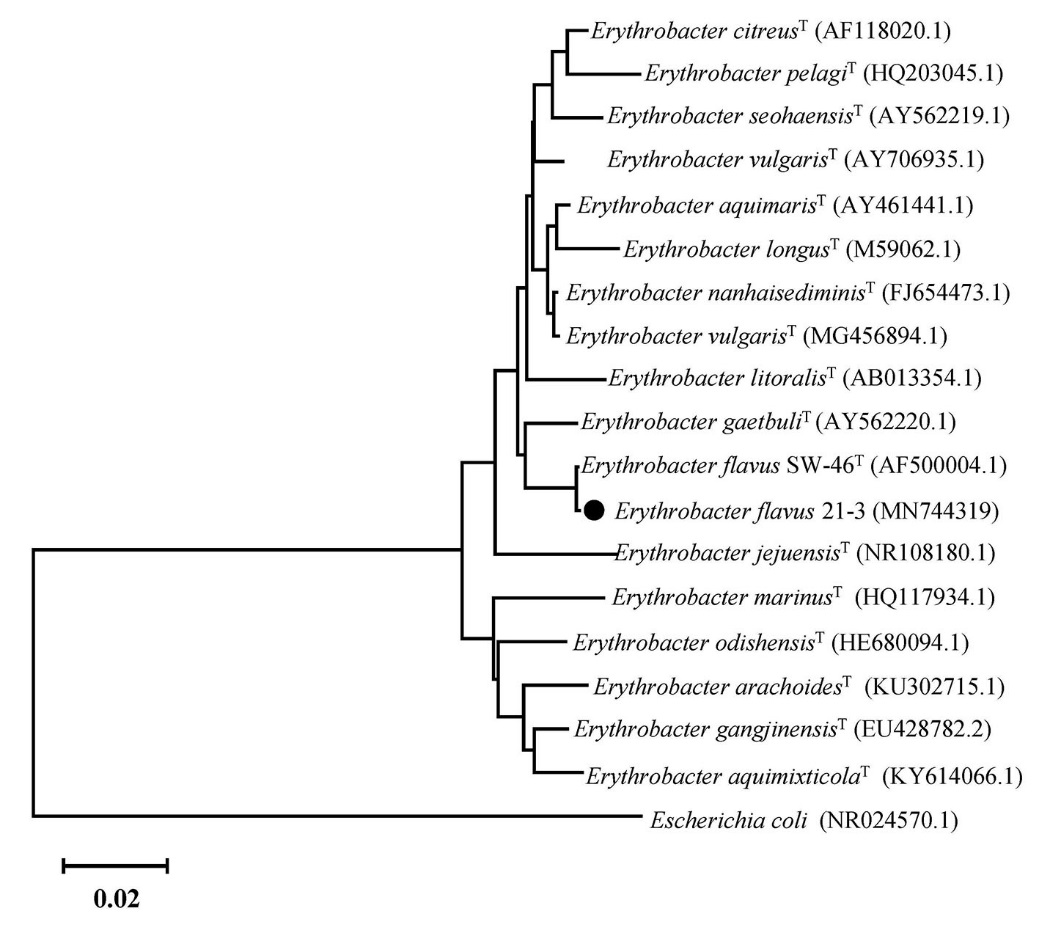
**

**Fig. S1** The consensus phylogenetic tree of *E. flavus* 21-3 with other related strains obtained from GenBank (accession numbers of 16S rRNA are indicated after the species name) constructed by the neighbor-joining method. The bootstrap support values 1000.

**
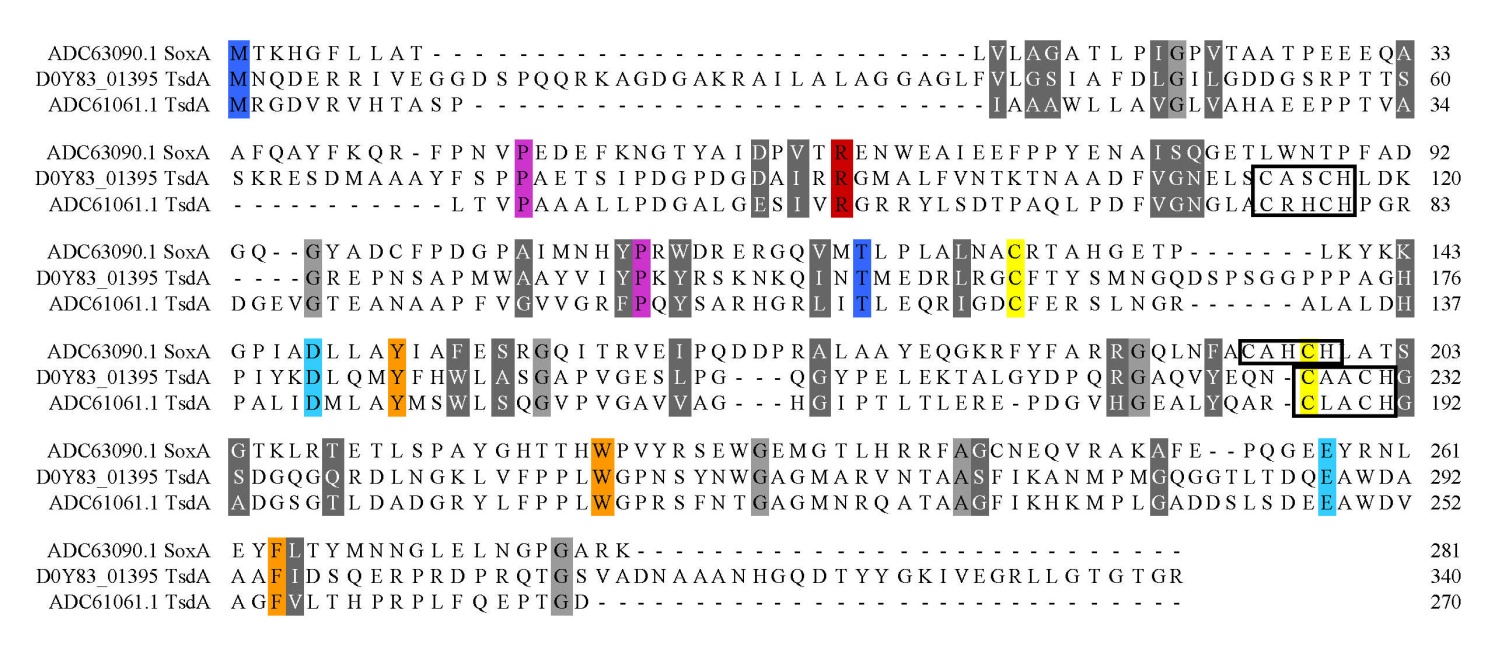
Fig. S2** Sequence alignment of TsdA from *E. flavus* 21-3 and *Allochromatium vinosum* DSM 180, and SoxA from *A.* *vinosum* DSM 180. Boxes showed the C××CH motif, and the conserved sites in all three sequences were labeled with the same color. The accession number of each protein is indicated before the protein name.

**
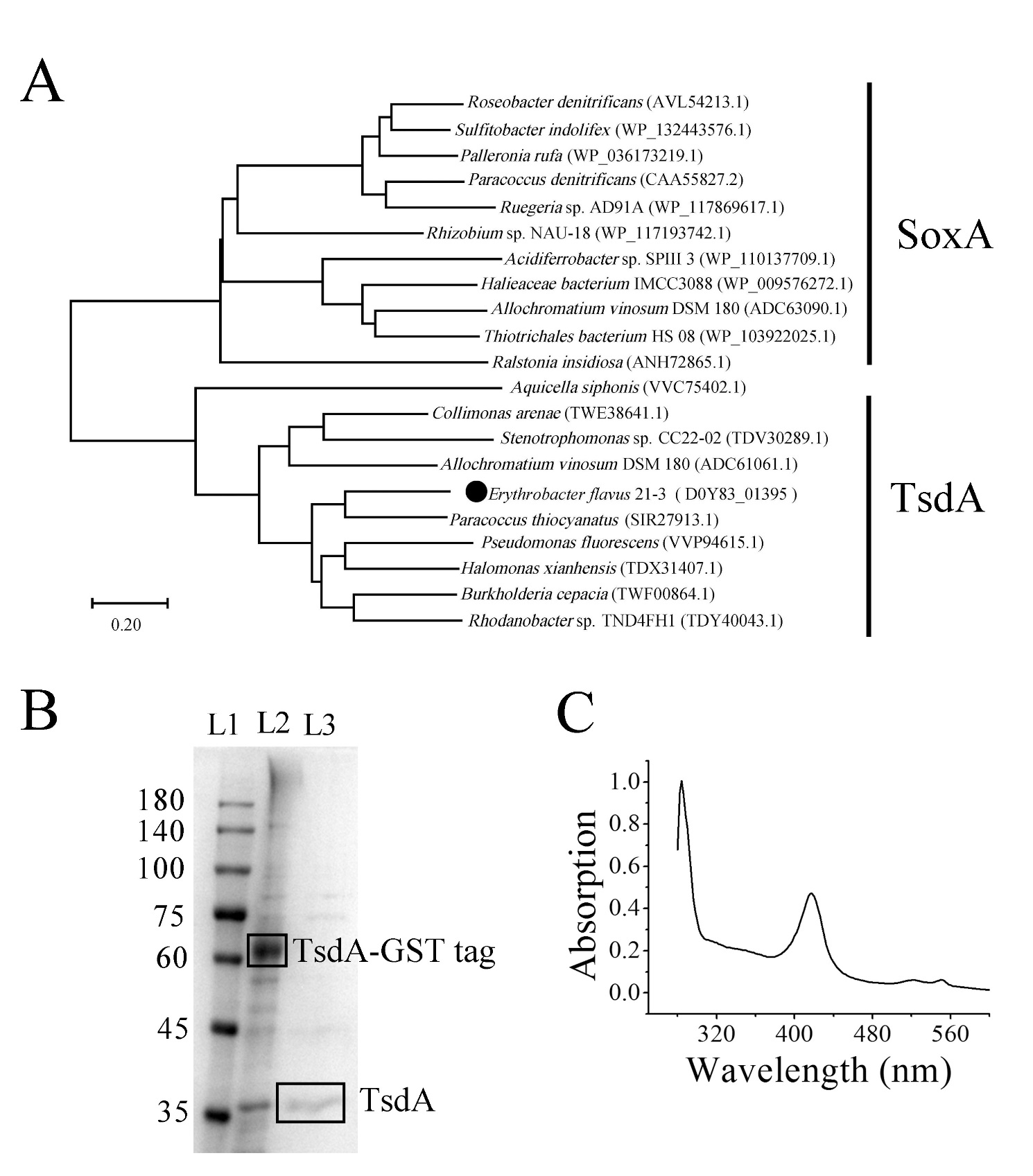
Fig. S3** Characterization of TsdA. (A) The consensus phylogenetic tree of TsdA in *E. flavus* 21-3 with other related TsdA and SoxA obtained from GenBank (accession numbers are indicated after the species name) constructed by the neighbor-joining method. The bootstrap support values 1000. (B) SDS-PAGE analysis of purified TsdA overexpressed in *E. coli*. L1, molecular weight marker. L2, expressed TsdA with a 26 KD GST tag. L3, TsdA after cleavage of GST tag by TEV protease. (C) UV-visible absorption spectrum of purified TsdA.

**
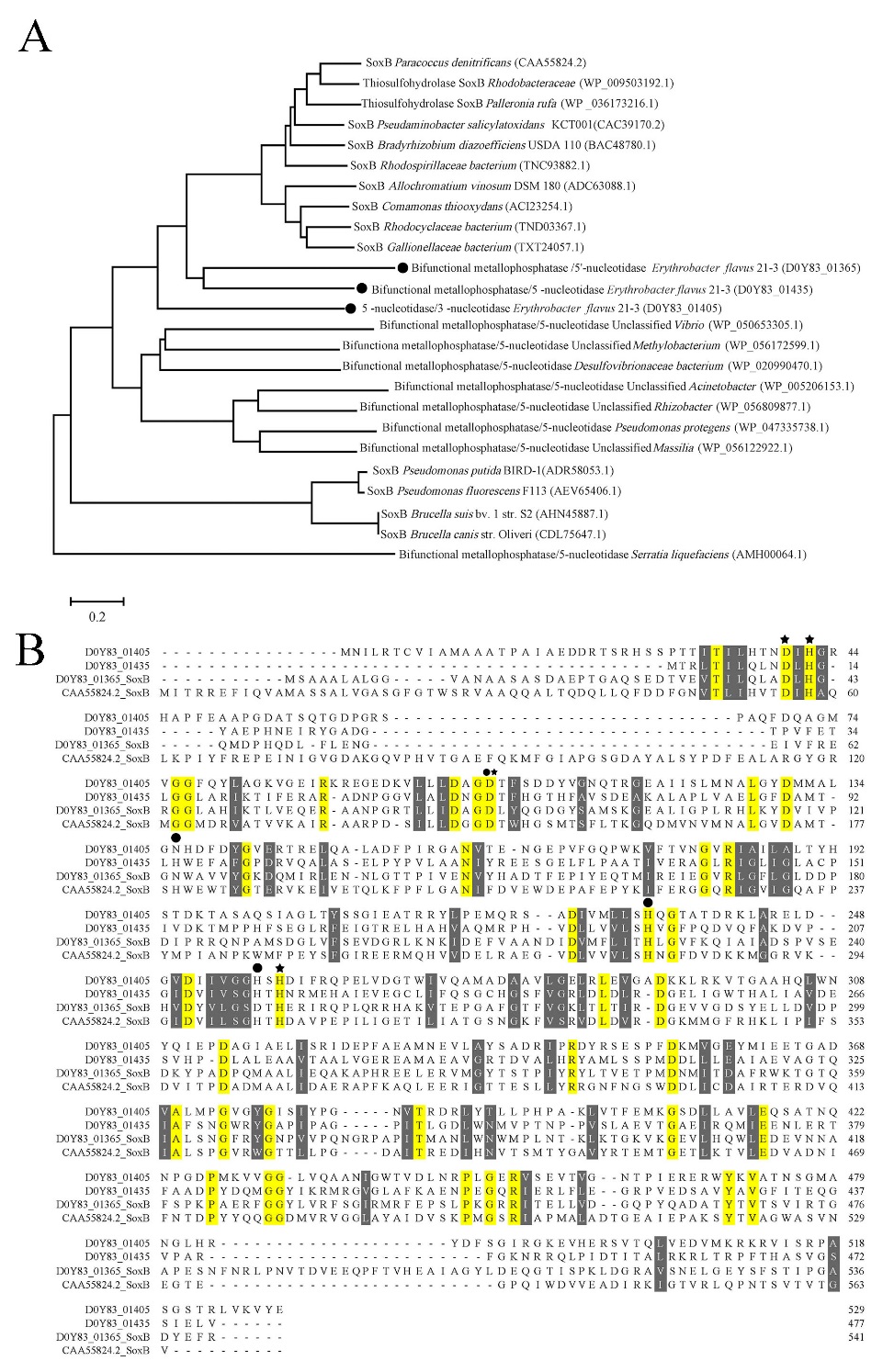
**

**Fig. S4** Similarity between *D0Y83_01365*, *D0Y83_01405* and *D0Y83_01435* encoding proteins and the typical thiosulfohydrolase CAA55824.2_SoxB. (A) The consensus phylogenetic tree of SoxB in *E. flavus* 21-3 with other related SoxBs obtained from GenBank (accession numbers are indicated after the proteins and species name) constructed by the neighbor-joining method. The bootstrap support values 1000. (B) Sequence alignment of SoxB candidates encoded by *D0Y83_01365*, *D0Y83_01405* and *D0Y83_01435* respectively in *E. flavus* 21-3 and the typical thiosulfohydrolase CAA55824.2_SoxB identified in *Paracoccus denitrificans*. The conserved sites in these sequences were labeled with yellow. Two ligands of metal sites were labeled with star and dot, respectively.

**
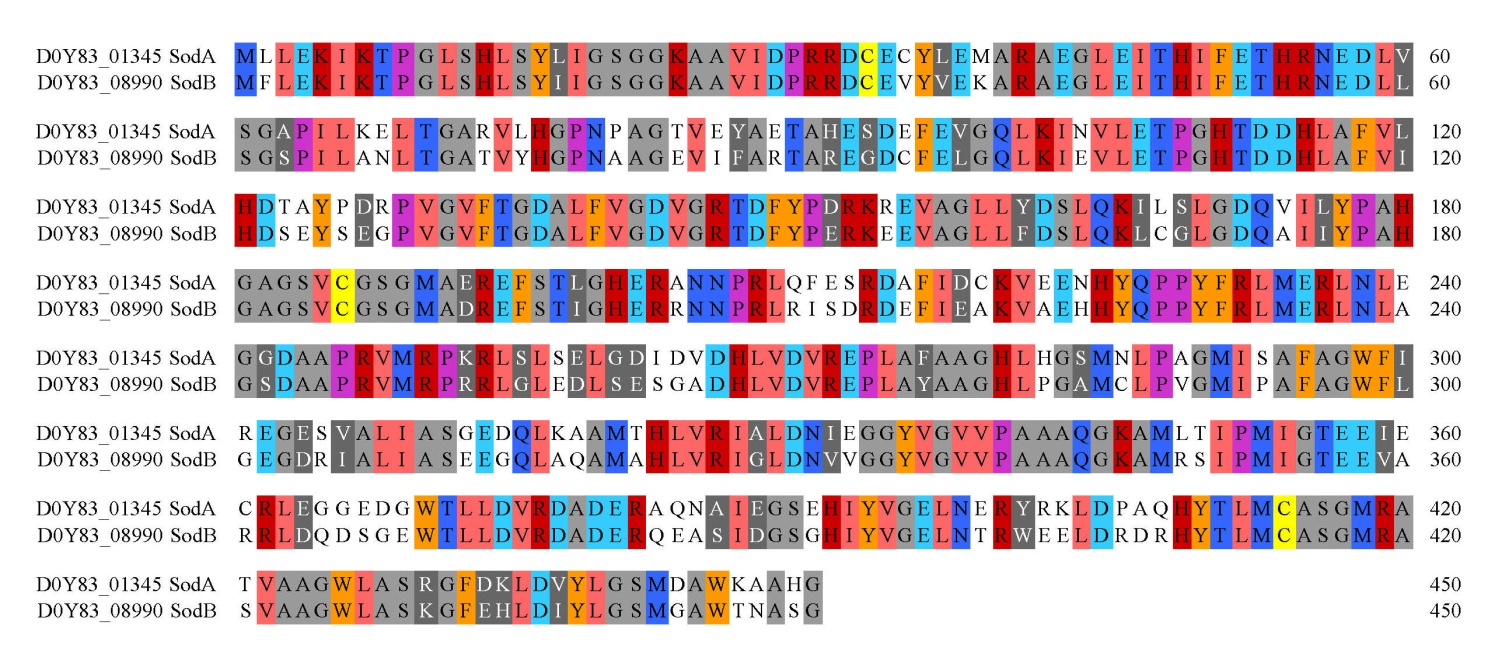
Fig. S5** Sequence alignment of SdoA (D0Y83_01345) and SdoB (D0Y83_08990) from *E. flavus* 21-3. The conserved sites in the two sequences were labeled with the same color.

**
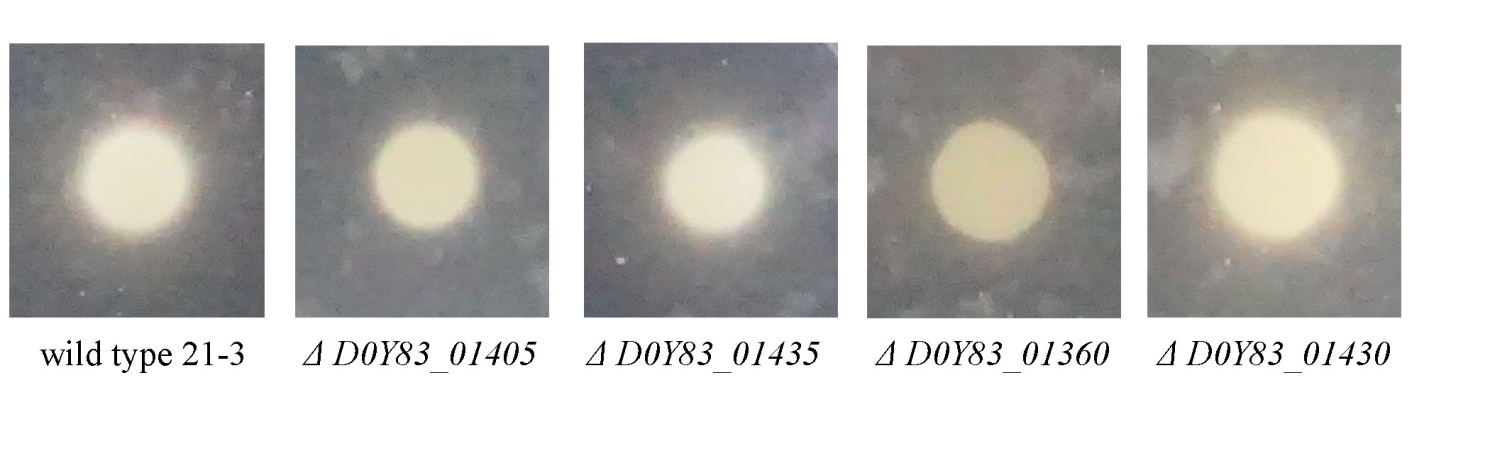
**

**Fig. S6** *E. flavus* 21-3 mutant strains *ΔD0Y83_01405*, *ΔD0Y83_01435,* *ΔD0Y83_01360* and *ΔD0Y83_01430* still produce ZVS when cultured with medium supplemented with 40 mM thiosulfate.

**
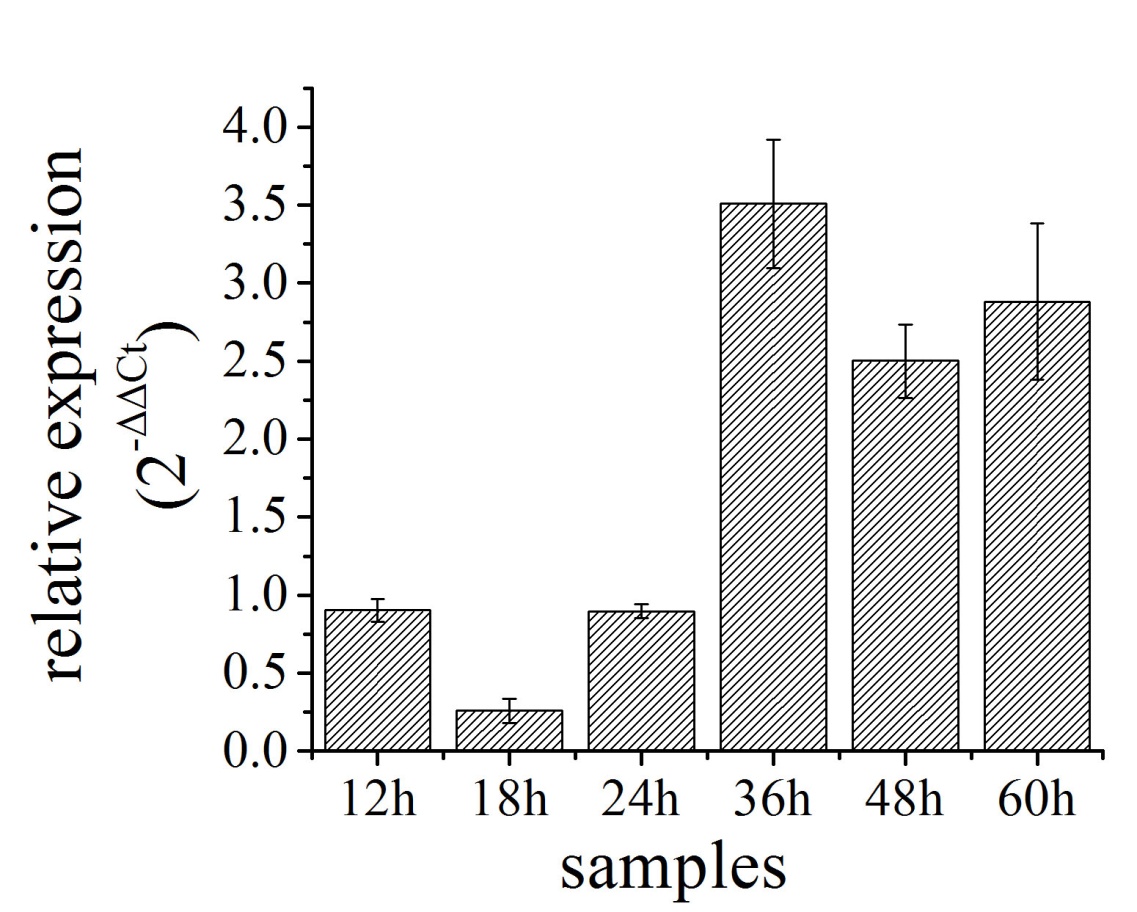
**

**Fig. S7** qRT-PCR analysis of the expression of *sdoB* in different time points in *E. flavus* 21-3 mutant *ΔsdoA* supplemented with 40 mM thiosulfate. All qRT-PCR runs were conducted with three biological and three technical replicates.

**
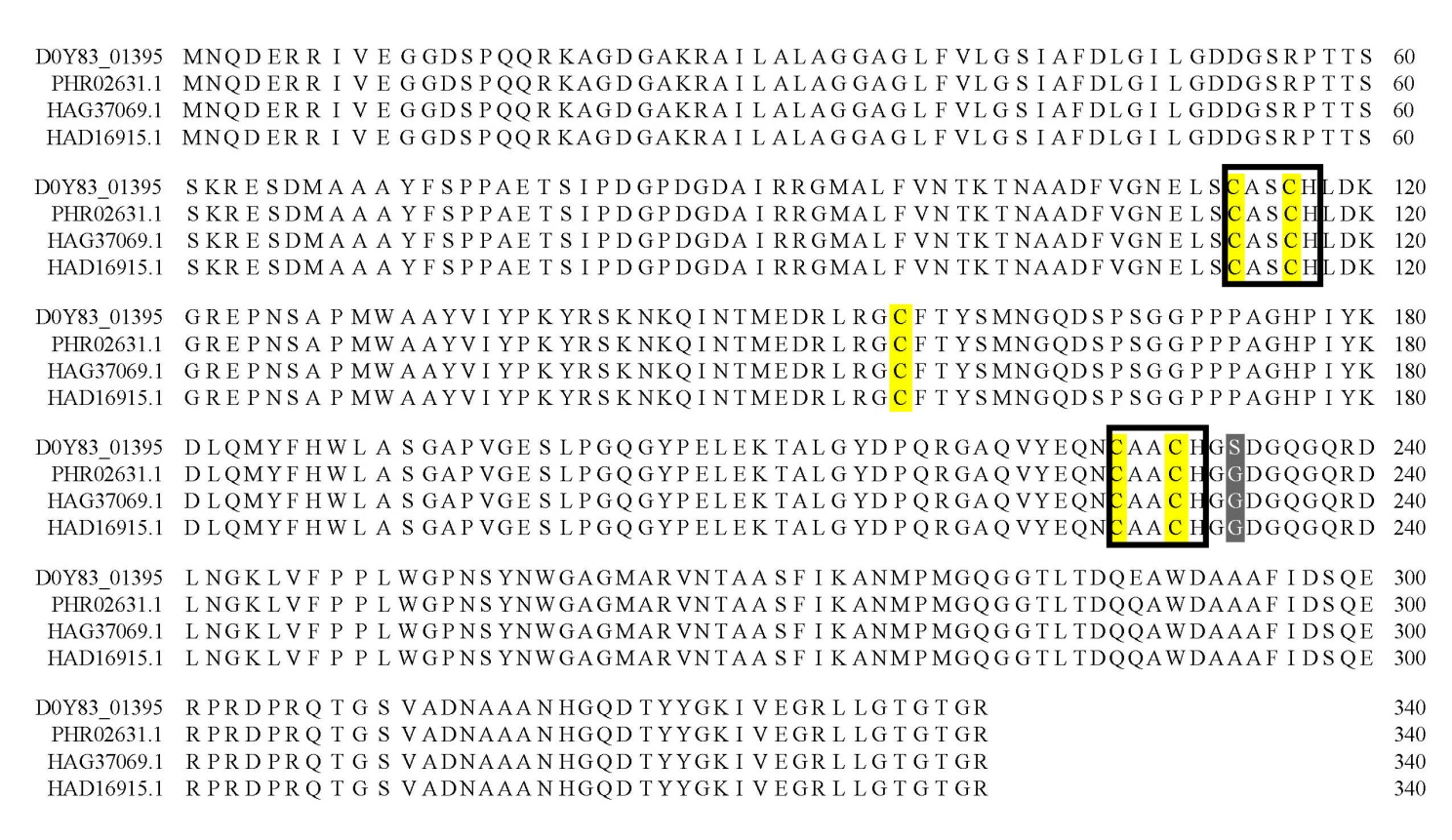
Fig. S8** Sequence alignment of TsdA from *E. flavus* 21-3 (D0Y83_01395), *Erythrobacter sp*. NORP101 (PHR02631.1), *Erythrobacter* sp. UBA9459 (HAG37069.1) and *Erythrobacter sp.* UBA9044 (HAD16915.1). Boxes show the C××CH motif. Accession numbers are indicated before the amino acid sequence in each line.

**
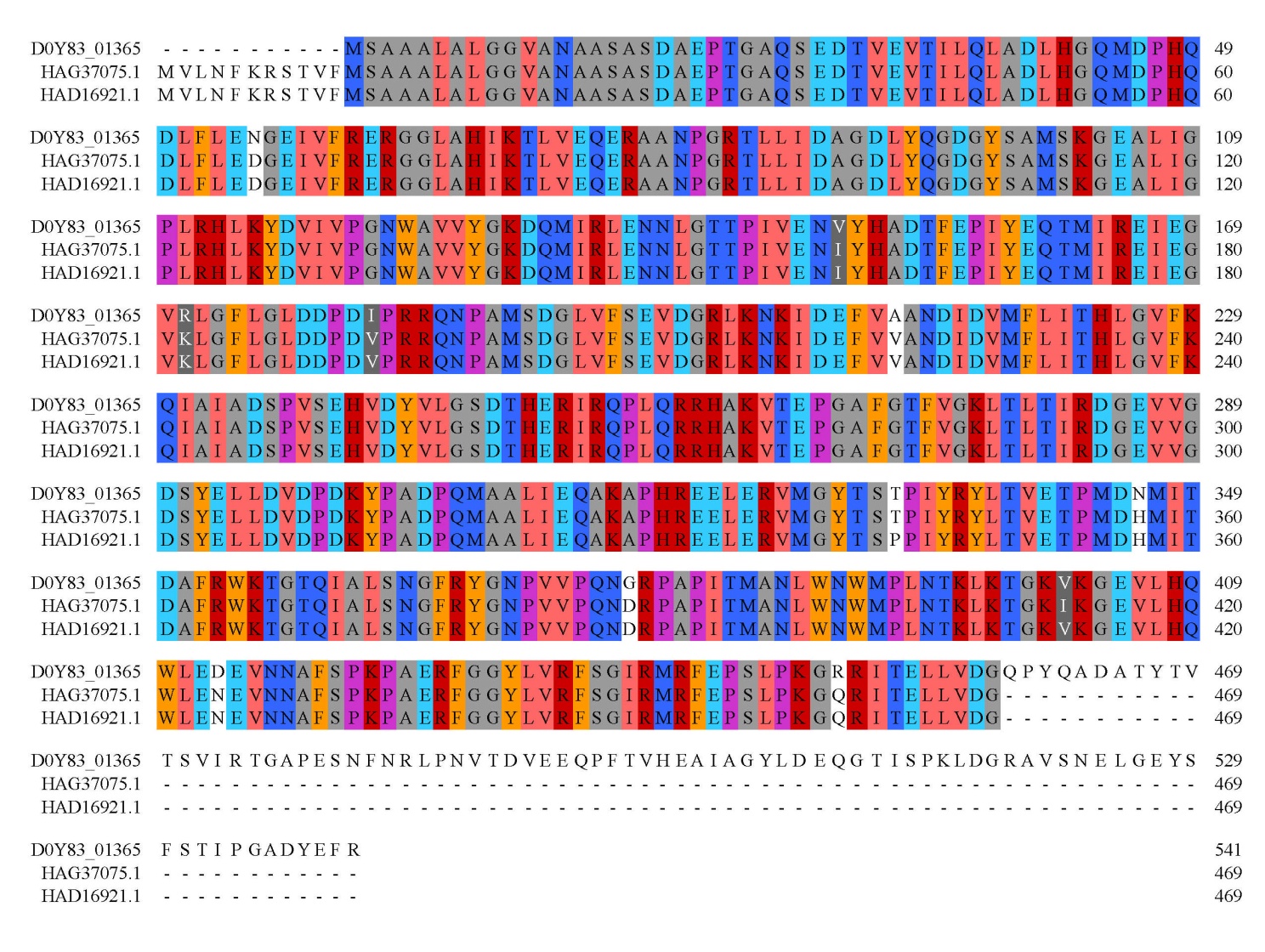
Fig. S9** Sequence alignment of SoxB from *E. flavus* 21-3 (D0Y83_01365), *Erythrobacter* sp. UBA9459 (HAG37075.1) and *Erythrobacter* sp. UBA9044 (HAD16921.1). The conserved sites in all three SoxB sequences are labeled with the same color. Accession numbers are indicated before the amino acid sequence in each line.

**Supplementary Table S1. Environmental and chemical parameters of the sampling site**

| Environmental and chemical parameters for sample site | pH | Temperature | Dissolved oxygen |
| --- | --- | --- | --- |
| Data | 7.68 | 3.75 °C | 3.07 mg/L |

**Supplementary Table S2. Strains, plasmids and primers used in this study**

| **Strains** | **Relevant genotype, description or sequence** | **Reference or source** |
| --- | --- | --- |
| *Erythrobacter flavus* 21-3 | wild type, isolated from sediment in deep sea clod seep | this work |
| *E. flavus* 21-3 *ΔtsdA* | Deletion of *tsdA* (D0Y83_01395) in *E. flavus* 21-3 | this work |
| *E. flavus* 21-3 *ΔsoxB* | Deletion of *soxB* (D0Y83_01365) in *E. flavus* 21-3 | this work |
| *E. flavus* 21-3 *ΔsdoA* | Deletion of *sdoA* (D0Y83_01345) in *E. flavus* 21-3 | this work |
| *E. flavus* 21-3 *ΔsdoB* | Deletion of *sdoB* (D0Y83_08990) in *E. flavus* 21-3 | this work |
| *E. flavus* 21-3 *ΔsdoAB* | Deletion of *sdoB* (D0Y83_08990) in *E. flavus* 21-3 *ΔsdoA* | this work |
| *E. flavus* 21-3 *ΔD0Y83_01360* | Deletion of D0Y83_01360 in *E. flavus* 21-3 | this work |
| *E. flavus* 21-3 *ΔD0Y83_01405* | Deletion of D0Y83_01405 in *E. flavus* 21-3 | this work |
| *E. flavus* 21-3 *ΔD0Y83_01430* | Deletion of D0Y83_01430 in *E. flavus* 21-3 | this work |
| *E. flavus* 21-3 *ΔD0Y83_01435* | Deletion of D0Y83_01435 in *E. flavus* 21-3 | this work |
| *Escherichia coli* DH5α | hsdR recA lacZYA ϕ80 lacZΔM15 | this work |
| *Escherichia coli* SY327 | F^−^ araD *Δ(lac-proAB)* argE(Am) rif nalA recA56 | [4] |
| *Escherichia coli* S17-1 | thi pro hsdr - hsdM + recA [ chr:: RP4-2-Tc::Mu-Km::Tn7] | [5] |
| **Plasmids** |  |  |
| pEX18Gm | 5.8 kb plasmid, Gm^R^, suicide vector | [6] |
| pEX-18Gm-KO-tsdA | Gm^R^, an overlap of 1750 bp inserted into pEX18Gm using *Eco*RⅠand *Bam*HⅠ | this work |
| pEX-18Gm-KO-soxB | Gm^R^, an overlap of 1743 bp inserted into pEX18Gm using *Eco*RⅠand *Bam*HⅠ | this work |
| pEX-18Gm-KO-sdoA | Gm^R^, an overlap of 1743 bp inserted into pEX18Gm using *Eco*RⅠand *Hin*d Ⅲ | this work |
| pEX-18Gm-KO-sdoB | Gm^R^, an overlap of 1959 bp inserted into pEX18Gm using *Hin*d Ⅲ and *Bam*HⅠ | this work |
| pEX-18Gm-KO-1435 | Gm^R^, an overlap of 1560 bp inserted into pEX18Gm using *Hin*d Ⅲand *Bam*HⅠ | this work |
| pEX-18Gm-KO-1405 | Gm^R^, an overlap of 1819 bp inserted into pEX18Gm using *Eco*RⅠand *Hin*d Ⅲ | this work |
| pEX-18Gm-KO-1360 | Gm^R^, an overlap of 1277 bp inserted into pEX18Gm using *Eco*RⅠand *Hin*d Ⅲ | this work |
| pEX-18Gm-KO-1430 | Gm^R^, an overlap of 735 bp inserted into pEX18Gm using *Eco*RⅠand *Bam*HⅠ | this work |
| 2GT | Amp^R^, His_6_-GST-TEV-yORF | [1] |
| 2GT-tsdA | *tsdA* gene cloned into 2GT | This work |
| pEC86 | Cm^R^, product from pEC66 and pACYC184 with *E. coli ccm*ABCDEFGH genes | [7] |
| **Primers (5’-3’)** |  |  |
| tsdA-up_F | CCGGAATTCCGGCAGAGTGGGCGATGAAACCT (*Eco*RⅠ) |  |
| tsdA-up_R | CGCGAACCTTTTCCGGTTCTCTTCACCTCGTCGGCTG |  |
| tsdA-down_F | CAGCCGACGAGGTGAAGAGAACCGGAAAAGGTTCGCG |  |
| tsdA-down_R | CGCGGATCCGCGGAGCTGGCGATCGAGATTGA (*Bam*HⅠ) |  |
| soxB-up_F | CCGGAATTCCGG GAGAACCCATCCTCGCTCTG (*Eco*RⅠ) |  |
| soxB-up_R | TGGCGCCCAATCAGAGGATAGCCGGTCGATCAACCCAACA |  |
| soxB-down_F | TGTTGGGTTGATCGACCGGCTATCCTCTGATTGGGCGCCA |  |
| soxB-down_R | CGCGGATCCGCG GCGCGGTGAAAGAGATTGAC (*Bam*HⅠ) |  |
| D0Y83_01435up_F | CGCGGATCCGCG GTCAACGGAATGGCTTTGGG (*Bam*HⅠ) |  |
| D0Y83_01435up_R | TAGCCGTCGGCGGAACGCCGGGTCTCGACCTTTCAGTTCT |  |
| D0Y83_01435down_F | AGAACTGAAAGGTCGAGACCCGGCGTTCCGCCGACGGCTA |  |
| D0Y83_01435down_R | CCCAAGCTTGGG GGTTGCTCCTCCTGATACGG (*Hin*d Ⅲ) |  |
| D0Y83_01405up_F | CCGGAATTC AAATGCGGTCTGCGTATTGC (*Eco*RⅠ) |  |
| D0Y83_01405up_R | AATCTGAAAGGTATTTGCCAGGCAGACAATGCGCGGCTCG |  |
| D0Y83_01405down_F | CGAGCCGCGCATTGTCTGCCTGGCAAATACCTTTCAGATT |  |
| D0Y83_01405down_R | CCCAAGCTT GGTGAAAGGCGATTTCTGGC (*Hin*d Ⅲ) |  |
| SdoA-up_F | CCGGAATTCCGG GGACGGTTCATCGACCTGTT (*Eco*RⅠ) |  |
| SdoA-up_R | TGTTCTTGAAGGAGGCACGACCAGACCAGGCAGCGTTC |  |
| SdoA-down_F | GAACGCTGCCTGGTCTGGTCGTGCCTCCTTCAAGAACA |  |
| SdoA-down_R | CCCAAGCTTGGG GAAGGAGATGGTTTCGGGCA (*Hin*d Ⅲ) |  |
| SdoB-up_F | CGCGGATCCGCG CTCCATACGCCGGGACATAC (*Bam*HⅠ) |  |
| SdoB-up_R | GCAACAGACAAGGAAGTCGCAGCGCCAAAATCTTGAAAAG |  |
| SdoB-down_F | CTTTTCAAGATTTTGGCGCTGCGACTTCCTTGTCTGTTGC |  |
| SdoB-down_R | CCCAAGCTTGGG GGCTATACCACCTGTCCAGC (*Hin*d Ⅲ) |  |
| D0Y83_01360up_F | CCGGAATTCCGG CGAGTTGGAGGTCGAAACCA (*Eco*RⅠ) |  |
| D0Y83_01360up_R | CACAAGGAAAGACAACGATTCTAATGCGGGGG |  |
| D0Y83_01360down_F | GGCCCCCCCCCGCATTAGAATCGTTGTCTTTCCTTGTG |  |
| D0Y83_01360down_R | CCCAAGCTTGGG CTCCGGTATCAGAATGCGCT (*Hin*d Ⅲ) |  |
| D0Y83_01430up_F | CCGGAATTCCGG AGTGTCCCTTCTGTCGCAAG (*Eco*RⅠ) |  |
| D0Y83_01430up_R | CCATCCCGGGCGAGGAGGGGGATTATGCCTCCTTG |  |
| D0Y83_01430down_F | CAAGGAGGCATAATCCCCCTCCTCGCCCGGGATGG |  |
| D0Y83_01430down_R | CGCGGATCCGCG GATCGTCTTGATGCGGGCTA (*Bam*HⅠ) |  |
| GTtsdA_F | TACTTCCAATCCAAT ATGAACCAGGATGAACGCCG |  |
| GTtsdA_R | TTATCCACTTCCAAT GCGCCCCGTGCCGGTCCCCA |  |

**Supplementary Table S3. General features of *E. flavus* 21-3 genome**

| Accession number | CP032228 |
| --- | --- |
| assembly size (bp) | 3.2 M |
| G + C content (%) | 63.99% |
| Genes (total) | 3,143 |
| CDS (total) | 3,085 |
| Genes (coding) | 3,034 |
| CDS (coding) | 3,034 |
| 5S rRNA genes | 2 |
| 16S rRNA genes | 2 |
| 23S rRNA genes | 2 |
| tRNA genes | 48 |
| nc RNAs | 4 |
| Pseudo Genes (total) | 51 |

**Supplementary Table S4. Genes related to sulfur metabolism in the genome of *E. flavus* 21-3**

| **locus_tag** | **protein name** | **nearest neighbor** | **nearest sequence** |
| --- | --- | --- | --- |
| D0Y83_03955 | aryl-sulfate sulfotransferase | *Erythrobacter flavus* | BBI19651.1 |
| D0Y83_01785 | cysteine desulfurase | *Sphingomonadales* | WP_060703634.1 |
| D0Y83_03205 | cysteine desulfurase | *Erythrobacter flavus* | WP_130585725 |
| D0Y83_07475 | cysteine desulfurase | *Erythrobacter sp.* EhN03 | WP_067469224 |
| D0Y83_02730 | disulfide bond formation protein B | *Erythrobacter* | WP_067465134.1 |
| D0Y83_08685 | DsbE family thiol:disulfide interchange protein | *unclassified Erythrobacter* | WP_067469791.1 |
| D0Y83_09400 | glutathione-dependentdisulfide-bond oxidoreductase | *Erythrobacter sp.* EhN03 | WP_067467395.1 |
| D0Y83_13155 | glutathione-disulfide reductase | *Erythrobacter* | WP_067507593.1 |
| D0Y83_14100 | nitrite/sulfite reductase | *Erythrobacter sp.* EhN03 | WP_067468017.1 |
| D0Y83_14090 | phosphoadenylyl-sulfate reductase | *Erythrobacter sp.* HI0020 | WP_067502430.1 |
| D0Y83_13480 | protein-disulfide isomerase | *unclassified Erythrobacter* | WP_067499890.1 |
| D0Y83_13485 | protein-disulfide isomerase | *Erythrobacter* | WP_067464147.1 |
| D0Y83_07275 | rhodanese-related sulfurtransferase | *Erythrobacter sp.* EhN03 | WP_067469121.1 |
| D0Y83_15325 | sulfate adenylyltransferase subunit CysD | *Erythrobacter sp.* HI0020 | WP_067504020.1 |
| D0Y83_15330 | sulfate adenylyltransferase subunit CysN | *Erythrobacter* | WP_067672783.1 |
| D0Y83_15260 | sulfate permease | *Erythrobacter sp.* HI00D59 | WP_067676339.1 |
| D0Y83_01935 | sulfite exporter TauE/SafE family protein | *Erythrobacter flavus* | WP_130585560.1 |
| D0Y83_03890 | sulfite exporter TauE/SafE family protein | *Erythrobacter sp.* HI0020 | WP_067501194.1 |
| D0Y83_06695 | sulfite exporter TauE/SafE family protein | *Erythrobacter* | WP_067462700.1 |
| D0Y83_08965 | sulfite exporter TauE/SafE family protein | *Sphingomonadales* | WP_010412736.1 |
| D0Y83_09645 | sulfite exporter TauE/SafE family protein | *Erythrobacter sp.* | HCS18035.1 |
| D0Y83_15085 | sulfite oxidase subunit YedZ | *Erythrobacter sp.* EhN03 | WP_067467827.1 |
| D0Y83_05760 | sulfotransferase | *Erythrobacter sp.* EhN03 | WP_067462121.1 |
| D0Y83_06435 | sulfotransferase | *Erythrobacter sp.* HI00D59 | WP_082834718.1 |
| D0Y83_13765 | sulfotransferase | *Erythrobacter flavus* | WP_094062747.1 |
| D0Y83_04680 | sulfotransferase family protein | *Erythrobacter flavus* | WP_130585871.1 |
| D0Y83_06465 | sulfotransferase family protein | *Erythrobacter sp.* HI00D59 | WP_067674342.1 |
| D0Y83_10610 | sulfotransferase family protein | *Erythrobacter sp.* | HCS17231.1 |
| D0Y83_03135 | sulfur carrier protein ThiS | *Erythrobacter sp.* HI00D59 | KZX52404.1 |
| D0Y83_14125 | sulfurtransferase | *Erythrobacter* | WP_067468026.1 |
| D0Y83_01430 | thiol:disulfide interchange protein | *Altererythrobacter sp. Ery1* | WP_119511759.1 |
| D0Y83_13970 | thiol:disulfide interchange protein | *Erythrobacter flavus* | WP_130586980.1 |
| D0Y83_13250 | thiol:disulfide oxidoreductase | *Erythrobacter sp.* HI00D59 | WP_067672469.1 |
| D0Y83_07440 | thioredoxin-disulfide reductase | *Erythrobacter sp.* EhN03 | WP_067469206.1 |
| D0Y83_13305 | thioredoxin-disulfide reductase | *Erythrobacter flavus* | WP_094062797.1 |

**Supplementary Table S5.** **Co-existence of TsdA and SoxB homologs in the published genomes**

| **Proteobacteria** | | |
| --- | --- | --- |
| **Order/family** | **Species** | **co-occurence of *soxYZ*** |
| Alphaproteobacteria |  |  |
| Rhizobiales |  |  |
| Bradyrhizobiaceae | *Bosea sp.* AS-1 | NO MATCH |
|  | *Bradyrhizobium sp.* SK17 | complete |
|  | *Nitrobacter hamburgensis* X14 | complete |
| Methylobacteriaceae | *Methylobacterium sp.* C1 | NO MATCH |
|  | *Methylorubrum extorquens* AM1 | NO MATCH |
| Rhodobacterales |  |  |
| Rhodobacteraceae | *Defluviimonas alba cai42* | NO MATCH |
|  | *Salipiger profundus J*LT2016 | Incomplete |
|  | *Thioclava nitratireducens* 25B10_4 | Incomplete |
| Rhodospirillales |  |  |
| Acetobacteraceae | *Acidiphilium multivorum* AIU301 | complete |
| Rhodospirillaceae | *Azospirillum thiophilum* BV-S | complete |
| Rhodospirillales |  |  |
| Rhodospirillaceae | *Indioceanicola profundi* | NO MATCH |
| Betaproteobacteria |  |  |
| Burkholderiales |  |  |
| Alcaligenaceae | *Tetrathiobacter kashmirensis* WT001 | Incomplete |
|  | *Achromobacter xylosoxidans* A8 | complete |
|  | *Pusillimonas sp.* JR1/69-2-13 | complete |
| Burkholderiaceae | *Cupriavidus basilensis* 4G11 | complete |
|  | *Cupriavidus metallidurans* CH34 | complete |
|  | *Cupriavidus metallidurans* Ni-2 | complete |
|  | *Cupriavidus pinatubonensis* JMP134 | complete |
|  | *Cupriavidus sp.* USMAA1020 | complete |
|  | *Cupriavidus sp.* USMAA2-4 | complete |
|  | *Cupriavidus sp.* USMAHM13 | complete |
|  | *Pandoraea faecigallinarum* DSM 23572 | complete |
|  | *Polynucleobacter asymbioticus* P1-4-10KL | complete |
|  | *Polynucleobacter asymbioticus* P1-Kol8 | complete |
|  | *Polynucleobacter asymbioticus* QLW-P1DMWA-1 | complete |
|  | *Polynucleobacter asymbioticus* Recht-1 | complete |
|  | *Polynucleobacter asymbioticus* Tro-7-1-4 | complete |
|  | *Polynucleobacter asymbioticus* Tro-8-2-9 | complete |
|  | *Ralstonia insidiosa* ATCC 49129 | complete |
|  | *Ralstonia insidiosa* FC1138 | complete |
|  | *Ralstonia mannitolilytica* SN82F48 | complete |
|  | *Ralstonia mannitolilytica* SN83A39 | complete |
|  | *Ralstonia pickettii* 12D | complete |
|  | *Ralstonia pickettii* FDAARGOS_410 | complete |
| Comamonadaceae | *Comamonas testosteroni* P19 | Incomplete |
|  | *Comamonas testosteroni* TK102 | Incomplete |
|  | *Comamonas testosteroni sv.* Ba CNB-1 | complete |
| Oxalobacteraceae | *Massilia alkalitolerans* DSM 17462 | Incomplete |
|  | *Thiomonas arsenitoxydans* 3As | complete |
|  | *Thiomonas intermedia* ATCC 15466 | complete |
|  | *Thiomonas intermedia* K12 | complete |
|  | *Thiomonas sp.* X19 | complete |
| Nitrosomonadales |  |  |
| Gallionellaceae | *Sideroxydans lithotrophicus* ES-1 | complete |
|  | *Sulfuriferula sp.* AH1 | complete |
| Thiobacillaceae | *Thiobacillus denitrificans* ATCC 25259 | complete |
| Gammaproteobacteria |  |  |
| Alteromonadales |  |  |
| Alteromonadaceae | *Marinobacter salinus* Hb8 | complete |
| Chromatiales |  |  |
| Chromatiaceae | *Chromatiaceae bacterium* 2141T.STBD.0c.01a | complete |
|  | *Allochromatium vinosum* DSM 180 | complete |
|  | *Marichromatium purpuratum* 984 | complete |
|  | *Thiocystis violascens 611,* DSM 198 | complete |
| Ectothiorhodospiraceae | *Thioalkalivibrio versutus* D301 | Incomplete |
| Granulosicoccaceae | *Sulfuriflexus mobilis* aks1 | complete |
| Halothiobacillaceae | *Halothiobacillus neapolitanus c2,* ATCC 23641 | Incomplete |
|  | *Halothiobacillus sp.* LS2 |  |
| **Bacteroidetes** | | |
| Hymenobacteraceae | *Hymenobacter* sp. PAMC 26554 | NO MATCH |
| Flavobacteriaceae | *Maribacter* sp. T28 | NO MATCH |

**Supplementary Table S6.** **Co-existence of TsdA and SoxB homologs**

**in the published metagenomes**

| Sample ID | Sample description | Number of homologs | |
| --- | --- | --- | --- |
|  |  | TsdA | SoxB |
| 2001200001 | Soil microbial communities from Waseca County, Minnesota, USA - Sample 10150 | 3 | 6 |
| 2001200002 | Fossil microbial communities from Whale Fall, Santa Cruz Basin, Pacific Ocean - Rib Bone Sample | 1 | 1 |
| 2001200003 | Fossil microbial communities from Whale Fall, Santa Cruz Basin - Microbial Mat Sample | 1 | 2 |
| 2001200004 | Fossil microbial communities from Whale Fall, West Antarctic Peninsula - Bone | 1 | 2 |
| 2189573024 | Echo Passage metagenome | 1 | 7 |
| 3300003691 | Combined assembly of microbial communities in oil-polluted sediment from the Gulf of Mexico | 2 | 8 |
| 3300006093 | Marine microbial communities from the Eastern Tropical South Pacific Oxygen Minimum Zone, cruise NBP1315, 2013 - sample NBP189 | 1 | 9 |
| 3300006561 | Marine microbial communities from the Black Sea in Odessa region - Od_1 | 2 | 2 |
| 3300007113 | Seawater microbiome, Papua New Guinea CO2 seep, Upa-Upasina "bubble" site, Water-is | 3 | 31 |
| 3300007114 | Seawater microbiome, Papua New Guinea CO2 seep, Upa-Upasina "bubble", waterEBis4 | 4 | 37 |
| 3300007116 | Seawater microbiome, Papua New Guinea CO2 seep, Upa-Upasina "bubble" site, waterEBis3 | 4 | 38 |
| 3300007144 | Seawater microbiome, Papua New Guinea CO2 seep, Upa-Upasina "control", waterEBic1 | 1 | 26 |
| 3300023187 | Activated sludge enriched bacterial communities from WWTP in Fort Collins, Colorado, USA ? OA | 4 | 9 |
| 3300023194 | Activated sludge enriched bacterial communities from WWTP in Fort Collins, Colorado, USA ? ML | 27 | 17 |
| 3300023200 | Activated sludge enriched bacterial communities from WWTP in Fort Collins, Colorado, USA ? LHP | 9 | 4 |
| 3300023201 | Activated sludge enriched bacterial communities from WWTP in Fort Collins, Colorado, USA ? PN | 19 | 24 |
| 3300023211 | Activated sludge enriched bacterial communities from WWTP in Fort Collins, Colorado, USA ? CA | 30 | 55 |
| 3300033002 | Soil microbial community from agricultural field in Dibrughar, Assam, India - D1 | 14 | 9 |

**References**

1. Sun C, Todorovic A, Querol-Audí J, Bai Y, Villa N, Snyder M, et al. Functional reconstitution of human eukaryotic translation initiation factor 3 (eIF3). Proc Natl Acad Sci USA. 2011; **108**: 20473-20478.

2. Arslan E, Schulz H, Zufferey R, Künzler P and Thöny Meyer L. Overproduction of the *Bradyrhizobium japonicum* *c*-type cytochrome subunits of the cbb3 oxidase in *Escherichia coli*. Biochem Biophys Res Commun. 1998; **251**: 744-747.

3. Tropea JE, Cherry S and Waugh DS. Expression and purification of soluble His_6_-tagged TEV protease. In: S. A. Doyle. High throughput protein expression and purification: Methods and Protocols. Totowa, NJ, Humana Press 2009. 297-307.

4. Goldberg I and Mekalanos JJ. Cloning of the *Vibrio cholerae* *recA* gene and construction of a *Vibrio cholerae recA* mutant. J Bacteriol. 1986; **165**: 715-722.

5. Schäfer A, Tauch A, Jäger W, Kalinowski J, Thierbach G and Pühler A. Small mobilizable multi-purpose cloning vectors derived from the *Escherichia coli* plasmids pK18 and pK19: selection of defined deletions in the chromosome of *Corynebacterium glutamicum*. Gene. 1994; **145**: 69-73.

6. Hoang TT, Karkhoff-Schweizer RR, Kutchma AJ and Schweizer HP. A broad-host-range Flp-FRT recombination system for site-specific excision of chromosomally-located DNA sequences: application for isolation of unmarked *Pseudomonas aeruginosa* mutants. Gene. 1998; **212**: 77-86.

7. Arslan E, Schulz H, Zufferey R, Künzler P, Thöny-Meyer L. Overproduction of the *Bradyrhizobium japonicum* *c*-Type cytochrome subunits of the cbb3 oxidase in *Escherichia coli*. Biochem Biophys Res Commun. 1998; **251**: 744-747.
